# Paradoxical implication of BAX/BAK in the persistence of tetraploid cells

**DOI:** 10.1038/s41419-021-04321-3

**Published:** 2021-11-01

**Authors:** Jiayin Deng, Lucía G. Gutiérrez, Gautier Stoll, Omar Motiño, Isabelle Martins, Lucía Núñez, José Manuel Bravo-San Pedro, Juliette Humeau, Chloé Bordenave, Juncheng Pan, Hélène Fohrer-Ting, Sylvie Souquere, Gerard Pierron, Claudio Hetz, Carlos Villalobos, Guido Kroemer, Laura Senovilla

**Affiliations:** 1 Centre de Recherche des Cordeliers, Sorbonne Université, Inserm, Université de Paris, Equipe 11 Labellisée par la Ligue Contre le Cancer, F-75006 Paris, France; 2 Metabolomics and Cell Biology Platforms, Gustave Roussy Comprehensive Cancer Institute, Villejuif, France; 3 Unidad de Excelencia Instituto de Biología y Genética Molecular (IBGM), Universidad de Valladolid – CSIC, Valladolid, Spain; 4 Facultad de Medicina, Departamento de Fisiología, Universidad Complutense de Madrid, Madrid, Spain; 5 Institute for Research in Immunology and Cancer (IRIC), Université de Montréal, Montreal, QC Canada H3C 3J7; 6 Department of Medicine, Université de Montréal, Montreal, QC Canada H3C 3J7; 7 Centre de Recherche des Cordeliers, Center for Histology, Cell Imaging and Cytometry (CHIC), Sorbonne Université, Inserm, Université de Paris, F-75006 Paris, France; 8 AMMICA-UMS3655, Gustave Roussy, 94800 Villejuif, France; 9 CNRS, UMR9196, Gustave Roussy Cancer Campus, Villejuif, France; 10 Faculty of Medicine, Biomedical Neuroscience Institute (BNI), University of Chile, Santiago, 8380453 Chile; 11 Center for Geroscience, Brain Health and Metabolism (GERO), Santiago, 7800003 Chile; 12 The Buck Institute for Research in Aging, Novato, CA 94945 USA; 13 Pôle de Biologie, Hopitâl Européen George Pompidou, AP-HP, Paris, France

**Keywords:** Senescence, Cell death, Cell growth

## Abstract

Pro-apoptotic multi-domain proteins of the BCL2 family such as BAX and BAK are well known for their important role in the induction of mitochondrial outer membrane permeabilization (MOMP), which is the rate-limiting step of the intrinsic pathway of apoptosis. Human or mouse cells lacking both BAX and BAK (due to a double knockout, DKO) are notoriously resistant to MOMP and cell death induction. Here we report the surprising finding that BAX/BAK DKO cells proliferate less than control cells expressing both BAX and BAK (or either BAX or BAK) when they are driven into tetraploidy by transient exposure to the microtubule inhibitor nocodazole. Mechanistically, in contrast to their BAX/BAK-sufficient controls, tetraploid DKO cells activate a senescent program, as indicated by the overexpression of several cyclin-dependent kinase inhibitors and the activation of β-galactosidase. Moreover, DKO cells manifest alterations in ionomycin-mobilizable endoplasmic reticulum (ER) Ca^2+^ stores and store-operated Ca^2+^ entry that are affected by tetraploidization. DKO cells manifested reduced expression of endogenous sarcoplasmic/endoplasmic reticulum Ca^2+^ ATPase 2a (Serca2a) and transfection-enforced reintroduction of Serca2a, or reintroduction of an ER-targeted variant of BAK into DKO cells reestablished the same pattern of Ca^2+^ fluxes as observed in BAX/BAK-sufficient control cells. Serca2a reexpression and ER-targeted BAK also abolished the tetraploidy-induced senescence of DKO cells, placing ER Ca^2+^ fluxes downstream of the regulation of senescence by BAX/BAK. In conclusion, it appears that BAX/BAK prevent the induction of a tetraploidization-associated senescence program. Speculatively, this may contribute to the low incidence of cancers in BAX/BAK DKO mice and explain why human cancers rarely lose the expression of both BAX and BAK.

## Introduction

BCL2 (B-cell lymphoma 2) family is known for their role in regulating the intrinsic (mitochondrial) pathway of apoptosis. According to their domain structure (organized around so-called BCL2 homology [BH] domains) and their functional contribution to mitochondrial outer membrane permeabilization (MOMP), which can be MOMP stimulatory (pro-apoptotic) or MOMP inhibitory (anti-apoptotic), the BCL2 proteins are classified into a multi-domain anti-apoptotic subfamily (BCL2 itself, BCL1L1, BCLW, and MCL1, which all have the full set of BH1 + BH2 + BH3 + BH4 domains), a multi-domain pro-apoptotic subfamily (BCL2-associated X protein (BAX), BAK, and BOK, which all have BH1 + BH2 + BH3 domains but lack BH4), and the so-called BH3-only proteins, which are all pro-apoptotic [1, 2]. Recent years have witnessed an ever-more-profound depth of insights into the dynamic structural alterations induced by the protein–protein interactions of these molecules [3, 4].

Although much work has helped to elucidate the detailed mechanisms through which BAX/BAK/BOK insert into the outer mitochondrial membrane to form higher-order oligomeric structure facilitating MOMP, how BCL2/BCL1L1/BCLW/MCL1 prevent MOMP, and how BH3-only proteins facilitate MOMP (either by the activation of BAX/BAK/BOK or the inhibition of BCL2/BCL1L1/BCLW/MCL1) [5, 6], it has become clear that these proteins have important functions in regulating other cellular functions [7]. Thus, BCL2 proteins have an impact on cellular metabolism (especially at the level of bioenergetics) [8], affect Ca^2+^ fluxes (especially at the level of the endoplasmic reticulum, ER) [9, 10], influence the advancement of the cell cycle [11], and regulate macroautophagy [12], just to mention a few among the “moonlighting” (non-MOMP-related) functions of the BCL2 family.

Members of the Bcl-2 family have been proposed to be regulators of intracellular Ca^2+^ signaling in cell survival and cell death by regulating Ca^2+^ transport systems located in the ER and mitochondria membranes, and at the plasma membrane [13]. It has been widely demonstrated that Ca^2+^ is involved in cell cycle progression [14–16]. Ca^2+^ controls the cell cycle mainly through the Ca^2+^/calmodulin complex that regulates the expression of cyclin-dependent protein kinases (CDKs), which, in turn, control the cell cycle. However, Ca^2+^ fluxes undergo alterations related to checkpoints found throughout the cell cycle [17, 18]. Errors occurring during the cell cycle are detected by these checkpoints leading to cell cycle arrest or even cell death [19]. The cell cycle arrest may be temporary or permanent, depending on whether the cellular machinery can repair the failure or not. Ca^2+^ signaling also participates in the re-entry of arrested cells (G0) into the cell cycle by the activation of transcription factors such as FOS, JUN, cyclic AMP-response element, and serum response element [16].

BAX and BAK localize at both the mitochondria and ER. Cells deficient for Bax, Bak (double knockout, DKO) display lowered steady-state ER Ca^2+^ concentrations ([Ca^2+^]_ER_) and secondarily decreased mitochondrial Ca^2+^ uptake [20]. DKO cells show reduced ER Ca^2+^ levels due to increased Ca^2+^ leakage and increased Ca^2+^ permeability due to the hyperphosphorylated state of the IP3R1. Members of the BCL2 family regulate IP3R1 phosphorylation, thereby controlling Ca^2+^ leakage from the ER. At the ER, BCL2 and IP3R1 interact, and this binding is increased in the absence of BAX and BAK [21]. On the other hand, expression of sarcoplasmic/ER Ca^2+^ ATPase 2a (Serca2a) restores [Ca^2+^]_ER_ and mitochondrial Ca^2+^ uptake in DKO cells reestablishing the mitochondrial pathway of apoptosis [22]. Moreover, DKO cells exclusively expressing BAK in the ER can undergo apoptosis independently of the canonical BAX, BAK-dependent mitochondrial apoptotic pathway [23]. Therefore, there is clear evidence that BAX and BAK somehow control Ca^2+^ flux. Multiple studies have investigated the functional role of BAX and BAK in Ca^2+^ control. In healthy cells, BAX and BAK adopt a monomer conformation in the mitochondrial outer membrane. Various stress signals induce a conformational change of these proteins by assembling into oligomeric complexes giving rise to pores of different sizes leading to MOMP during apoptosis [24–26]. In addition, ER stress inducers can lead to conformational changes and oligomerization of BAX and BAK on the ER, similar to the mitochondria [27]. BAX inhibitor-1 is a Ca^2+^ leak channel involved in the ER stress response [28–31]. It remains to be clarified whether these pores directly account for the passage of Ca^2+^ from the ER to the cytosol during apoptosis.

Of note, the DKO of BAX and BAK (and, more recently, the triple knockout [TKO] of BAX, BAK, and BOK) have been extensively studied to reveal the essential role of these proteins in the activation of MOMP and hence the intrinsic pathway of apoptosis [26, 32–34]. Nonetheless, it turned that BAX/BAK DKO cells do succumb in response to specific cell death stimuli, as exemplified by granzyme B [35], chelethrine [36], *N*-(3-oxododecanoyl)-homoserine lactone [37], or combined inhibition of glutathione and thioredoxin metabolism [38]. Although it can be argued that these cell death pathways do not rely on a physiological route towards MOMP, it appears that the cytoprotective action of BAX/BAK inactivation is not absolute. In this context, it appears intriguing that the phenotype of BAX/BAK and BAX/BAK/BOK-knockout mice is somehow deceptive. Indeed, although it has been speculated for long that apoptotic (developmental) cell death would play a major role in embryogenesis and fetal development [39–43], such mice are born with a clearly recognizable murine morphology (instead of forming amorphous cell masses) and—even though they contain supernumerary cells in multiple organ systems—rarely develop tumors [44, 45].

Intrigued by these observations, we tested a number of cell death-inducing chemotherapeutic drugs on BAX/BAK DKO cells. In this context, we observed that such cells do not tolerate tetraploidization in the sense that they undergo senescence in response to transient exposure to tetraploidy-inducing microtubule inhibitors. Here we describe this unsuspected phenotype and attempt to explain it in mechanistic terms.

## Materials and methods

### Materials

Unless otherwise indicated, the media and supplements used in cell culture were purchased from Gibco–Thermo Fisher Scientific (Waltham, MA, USA), the chemicals were purchased from Sigma-Aldrich (St Louis, MO, USA), and plasticware from Corning B.V. Life Sciences (Schiphol-Rijk, The Netherlands).

### Antibodies

Rabbit monoclonal antibody (Ab) against Bax (#2772) and rabbit polyclonal Ab against Bak (#3814) were purchased from Cell Signaling Technology (Danvers, MA, USA). Mouse monoclonal Ab against p27 ^Kip1^ (ab193379), rabbit monoclonal Abs against p16^INK4^ (ab51243), p21^Cip1^ (ab109199), p57 ^Kip2^ (ab75974), and β-actin–horseradish peroxidase (HRP) (ab49900) were purchased from Abcam (Cambridge, UK). Alexa fluor secondary Abs were purchased from Thermo Fisher. HRP Abs were purchased from Southern Biotechnologies Associates (Birmingham, UK).

### Cell lines and culture conditions

All cell lines were kept in culture at 37 °C under 5% of CO_2_. Wild type (WT) or genetically modified mouse embryonic fibroblasts (MEFs) for *Bax*^*−/−*^, *Bak*^*−/−*^, *Bax/Bak1*^*−/−*^ (DKO), *Pmaip1*^*−/−*^, *Bcl2l11*^*−/−*^, *Bbc3*^*−/−*^ [32], *Bbc3*^*−/−*^*Pmaip1*^*−/−*^, *Bcl2l11*^*−/−*^*Bbc3*^*−/−*^, *Bcl2l11*^*−/−*^*Bid*^*−/−*^ [46], DKO expressing rabbit Serca2a (DKO-Serca_oe_, [22], and DKO expressing Bak at the ER (DKO-Bak_ER_), provided by Hetz and colleagues [47], were cultured in Dulbecco’s modified Eagle’s medium (DMEM) containing 4500 mg L^−1^, 1 mM sodium pyruvate and supplemented with 10% fetal bovine serum (FBS), 10 mM HEPES buffer, 100 U mL^−1^ penicillin sodium, and 100 μg mL^−1^ streptomycin sulfate. Immortalized MEFs derived from C57Bl/6 WT or *Bax/Bak*^*−/−*^ mice, kindly provided by Strasser and colleagues [40], were cultured in DMEM containing 1000 mg L^−1^, 1 mM sodium pyruvate, and supplemented with 10% FBS, 50 mM 2-mercaptoethanol, 100 mM asparagine, and 100 U mL^−1^ penicillin sodium and 100 μg mL^−1^ streptomycin sulfate. Human colon carcinoma HCT116 cells (WT and DKO) were cultured in McCoy’s 5A (Modified) Medium supplemented with 10 % FBS, 1 mM sodium pyruvate, 10 mM HEPES, and 100 U mL^−1^ penicillin sodium and 100 μg mL^−1^ streptomycin sulfate.

### Generation of HCT116 DKO cells

HCT116 WT cells were purchased from the American Type Culture Collection (Manassas, VA, USA). HCT116 *BAX*^*−/−*^*BAK*^*−/−*^ (DKO) were generated from HCT116 *BAX*^*−/−*^ kindly provided by Vogelstein and colleagues [48, 49]. HCT116 *BAX*^*−/−*^ were transfected with Human BAK ZFN plasmid from Sigma-Aldrich (St Louis, MO, USA) following the manufacturer’s instructions. Transfected cells were cloned and characterized for BAX and BAK expression by immunoblotting.

### RNA isolation and reverse-transcription PCR

Total RNA was extracted and purified using RNeasy mini kit from QIAGEN (Hilden, Germany). Genomic DNA was eliminated by RNase-free DNase I from Invitrogen (Carlsbad, CA, USA). RNA quantification was performed using the NanoDrop2000 (Thermo Fisher Scientific). Reverse transcription (RT) of total RNA (2.5 μg) was performed using SuperScript™ IV VILO™ Master Mix (Invitrogen). Murine Serca2a (mSerca2a) RNA expression was calculated by RT-quantitative real-time PCR (qPCR). cDNA was amplified with specific mSerca2a probe (Mm01201431, Thermo Fisher Scientific) using Taqman Fast Master Mix (Applied Biosystems, Foster City, CA, USA). qPCR was performed on the StepOne Real-time PCR system (Applied Biosystems) according to the manufacturer’s instructions. Relative gene expression was calculated according to the 2^−ΔΔCt^ method, normalizing the level of mSerca2a to peptidylprolyl isomerase A mRNA, as endogenous control. Rabbit Serca2a (rbSerca2a) RNA expression was evaluated by RT semi-qPCR. rbSerca2a primers (Fwd: 5′-GGGCTGTCAACCAGGATA-3′; Rev: 5′-TGCAATGCAAATAAGGGA -3′; product length = 218 bp) were designed using Primer Premier 5 Design Program (Premier Biosoft International, San Francisco, CA, USA). β-Actin was used as internal control to normalized rbSerca2a expression (Fwd 5′-GCACCACACCTTCTACAATG -3′; Rev: 5′-TGCTTGCTGATCCACATCTG -3′; product length = 822). cDNA for rbSerca2a and β-actin were amplified using GoTaq® Hot Start Colorless Master Mix (Promega, Madison, WI, USA). PCR reactions were as follows: initial denaturation at 94 °C for 2 min, followed by 40 amplification cycles at 94 °C for 20 s during denaturation, annealing at 55 °C for 45 s, primer extension at 72 °C for 3 min, and final extension at 72 °C for 7 min. Amplified products were loaded on 2% agarose gels containing 10 μl of SYBR Green (Invitrogen). Images were acquired with the G:Box chemiluminescent system (GeneSys software) from Syngene (Cambridge, UK) and analyzed using the Image J software (National Institutes of Health, USA).

### Cytofluorometry

#### Cell death

Cells were seeded in 12-well plates. Next day, cells were treated with the following pharmacological agents: *cis*-diaminodicloroplatino(II) (10, 20, and 40 μM), oxaliplatin (10, 25, and 50 μM), carboplatin (1, 100, and 200 μM), nocodazole (Noco) (50, 100, and 200 nM), paclitaxel (150, 300, and 600 nM), rotenone (1, 5, and 10 μM), RO3280 (5, 25, and 50 μM), AZD1152 (0.5, 5, and 50 μM), or M2I-1 (25, 50, and 100 μM). After 48 h of treatment, apoptosis was measured in unfixed cells co-stained with 40 nM 3,3’dihexiloxalocarbocyanine iodide (DiOC_6_(3)) (Life Technologies, Carlsbad, CA, Estados Unidos) to quantify mitochondrial transmembrane potential (ΔΨm) plus 1 μg/mL propidium iodide (PI) (Life Technologies) to identify plasma membrane breakdown.

#### Senescence

Cells were seeded in 12-well plates. Next day, cells were treated with Noco 100 nM or cytochalasin D (CytD) 1.2 μM. After 48 h of treatment or 48 h of treatment + 4 days in drug-free culture medium, cells were labeled with the β-galactosidase substrate 5-Dodecanoylaminofluorescein Di-β-d-Galactopyranoside (C12FDG) (Thermo Fisher Scientific) plus 1 μg/mL PI and 10 μM Hoechst 33342 (Life Technologies).

Cytofluorometric determinations were performed by means of an Attune® LSRII Fortessa flow cytometer (BD Biosciences) (San Jose, CA, USA). Data analyses were carried out by using the FlowJo software, upon gating on the events characterized by normal forward scatter and side scatter values.

### Fluorescence imaging of cytosolic Ca^2+^

Cells were plated onto 12 mm ∅ coverslips previously coated with 0.01 mg/ml poly-l-lysine. Next day, cells were treated with Noco 100 nM. After 48 h of treatment, cells were loaded with 4 µM Fura2/AM (Life Technologies) for 1 h in external saline solution (ESS) containing (in mM): 145 NaCl, 5 KCl, 1 CaCl_2_, 1 MgCl_2_, 10 glucose, 10 Hepes/Na^+^ (pH 7.42). Cytosolic Ca^2+^ concentration ([Ca^2+^]_cyt_) was monitored as reported previously [50] by fluorescence imaging of cells using an OrcaER Hamamatsu digital camera (Hamamatsu Photonics, Hamamatsu, Japan) connected to an inverted Zeiss Axiovert microscope (Zeiss, Oberkochen, Germany), while being continuously perfused with ESS at 37 °C. Cells were epi-illuminated alternately at 340 and 380 nm using bandpass filters. Light emitted above 520 nm at both excitation lights was filtered by a dichroic mirror and collected every 5–10 s with a ×40, 1.4 numerical aperture, oil objective. To evaluate Ca^2+^ store content, cells were perfused with Ca^2+^-depleted ESS containing 0.5 mM of EGTA and 100 nM of the Ca^2+^ ionophore ionomycin. Intracellular free [Ca^2+^]_cyt_, rise in these conditions, reflects an estimation of the Ca^2+^ store content as previously reported [50]. For monitoring store-operated Ca^2+^ entry (SOCE), cells were treated with 10 μM cyclopiazonic acid for 10 min or 1 µM thapsigargin for 10 min in Ca^2+^-depleted ESS containing 0.5 mM of EGTA before the calcium imaging experiments, to deplete Ca^2+^ stores and to enable SOCE activation. Then, cells were perfused with 1 mM Ca^2+^-containing ESS to monitor [Ca^2+^]_cyt_ increase that is an estimation of SOCE.

### Clonogenicity assay

Cells were seeded in 175 cm^2^ flasks. After 24 h, cells were treated, or not, with Noco 100 nM or CytD 1.2 μM. After 48 h, cells were washed and kept in drug-free culture medium for 4 days more, followed by cloning cells. Prior to cloning, cells were labeled with 10 μM Hoechst 33342 for 1 h at 37 °C under 5% of CO_2_. Cells characterized by 2*n* or >4*n* DNA content were sorted on a FACs Influx cell sorter (BD Bioscience). Hoechst signal was read off the 405 nm laser, using the 460/50 nm detector. A control tube (cells without treatment) was used to position the gates for 2*n* and >4*n*. Then, 2*n* or >4*n* populations were sorted into 96-well plates (100 cells/well) and cultured for 2 weeks in drug-free culture medium. Then, wells showing cell proliferation were quantified.

### Clonogenic assay

Cells were seeded in 6-well plates at 1000, 2000, 5000, 10,000, 50,000, or 100,000 cells per well. After 24 h, cells were treated with Noco 100 nM for 48 h. Then, cells were washed and cultured in drug-free medium for 1 week (plates with 10,000, 50,000, and 100,000 cells per well) or 2 weeks (plates with 1000, 2000, and 5000 cells per well). After 1 or 2 weeks, supernatant was removed and 500 μl of Crystal Violet (Sigma) was added. Crystal violet was discarded after 10 min and wells were washed with deionized water. Colony area was quantified through Image J “ColonyArea” Plugin [51].

### Cell proliferation assay

Cells were prepared similarly to the clonogenicity assay. Cells characterized by >4*n* DNA content were sorted (300 cells/well in 96-well plates). Cell concentration was evaluated at 4, 6, 9, 11, and 14 days after sorting by flow cytometry (Attune® LSRII Fortessa).

### Immunoblotting

Cells were seeded into 75 or 175 cm^2^ flasks for control and treated conditions, respectively. After 24 h, cells were treated with Noco 100 nM. After 48 h, control cells were collected and treated cells washed and kept in drug-free culture medium for 4 days more. Then, only attached cells were collected, washed with phosphate-buffered saline, their pellets were lysed, and proteins quantified following standard procedures. At least 20 μg of protein were loaded on Bis-Tris 4–12% pre-cast gels (Thermo Fisher) and transferred to nitrocellulose or polyvinyldifluoride membranes (Millipore, Bedford, USA). Membranes were incubated for 1 h in Tris-buffered saline (TBS)-Tween 20 (0.05%) supplemented with 5% non-fat powdered milk or bovine serum albumin to block nonspecific binding sites. Primary Abs were incubated overnight at 4 °C, detected with the appropriate HRP-labeled secondary Abs, and revealed with Amersham ECL+ (GE Healthcare, Little Chalfont, UK). β-Actin was used as protein loading control.

### Statistical analysis

The experiments were repeated at least three times, which in our experience is optimal for obtaining significant results, with similar results. The specific *n* value for each experiment is specified in the corresponding figure. Outliers values were identified with the extreme studentized deviate method Grubb’s test by Graphpad. Given the variability in the size of the replicates, instead of using normal distribution test and equal variance test, we use two-sided (mixed) linear models. All statistical analysis from data without outliers were done within R environment [52]. We used the standard linear model inference (“lm” function) or the mixed model inference (nlme package) [53] and *p*-values estimations, according to the statistical question set in every figure legend. See Supplementary Table ST1 for details.

## Results

### Loss of clonogenicity of tetraploid Bax/Bak DKO cells

**Fig. 1:**
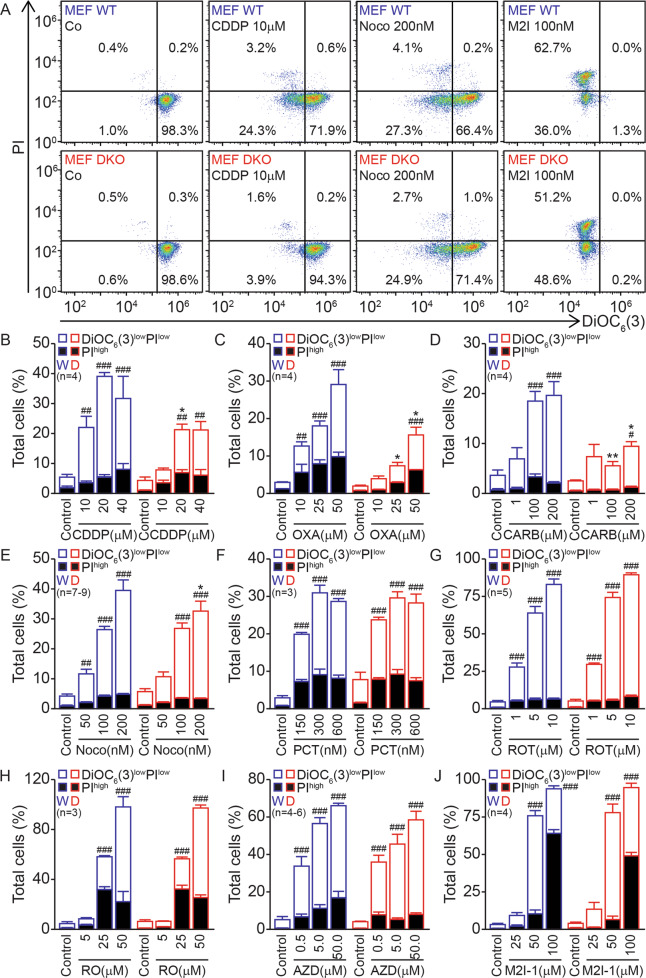
Bax/Bak DKO mouse embryonic fibroblasts (MEFs) are susceptible to killing by microtubular inhibitors. Wild type (WT) and DKO MEFs were cultured for 48 h in the absence (control, Co) or presence of 10, 20, and 40 μM *cis*-diaminodicloroplatino(II) (CDDP) (**A**, **B**); 10, 25, and 50 μM oxaliplatin (OXA) (**C**); 1, 100, and 200 μM carboplatin (CARB) (**D**); 50, 100, and 200 nM nocodazole (Noco) (**A**, **E**); 150, 300, and 600 nM paclitaxel (PCT) (**F**); 1, 5, and 10 μM rotenone (ROT) (**G**); 5, 25, and 50 μM RO3280 (RO) (**H**); 0.5, 5, and 50 μM AZD1152 (**I**); or 25, 50, and 100 μM M2I-1 (**A**, **J**). Then, cells were stained with the vital dye propidium iodide (PI) and the mitochondrial membrane potential (ΔΨ_m_)–sensitive dye DiOC_6_(3) to measure apoptotic cell death (**A**–**J**). **A** Illustrates representative dot plots of WT and DKO MEFs in the absence of any treatment (Co) or upon incubation with μM CDDP, 100 nM Noco, or 100 nM M2I-1. Numbers indicate the percentage of cells in each quadrant. In **B**–**J**, black columns represent the percentage of dead (PI^high^) cells and white columns represent the percentage of dying cells (DiOC_6_(3)^low^PI^low^). Error bars indicate SEM. Total columns (DiOC_6_(3)^low^PI^low^ + PI^high^) were compared by R software using standard linear model inference, “lm” function. #*p* < 0.05, ##*p* < 0.01, ###*p* < 0.001 treatment vs. control. *p < 0.05, **p < 0.01 treatment effect, DKO (“D”, in red) vs. WT (“W”, in blue).

As compared to normal WT controls, Bax/Bak DKO MEFs (Supplementary Fig. S1A) are highly resistant against a variety of anticancer drugs, including the DNA-damaging platinum-based chemotherapeutics cisplatin, oxaliplatin, and carboplatin, as determined by cell staining with the mitochondrial transmembrane (ΔΨ_m_)-sensitive fluorochrome DiOC_6_(3) and the vital dye PI to identify dying (DiOC_6_(3)^low^ PI^−^) and dead (DiOC_6_(3)^low^ PI^+^) cells (Fig. 1A–D). In contrast, Bax/Bak DKO are as susceptible as WT cells to killing by a variety of microtubular inhibitors such as Noco, paclitaxel, and high-dose rotenone (Fig. 1A, E–G), as well as by several agents that target the spindle checkpoint such as RO3380 (an inhibitor of polo-like kinase-1), AZD1152 (an inhibitor of aurora kinase B), and M2I-1 (an inhibitor of mitotic arrest deficient 2) (Fig. 1A, H–J). Similar results were obtained with BAX/BAK DKO human colon cancer HCT116 cells (Supplementary Fig. S1B), which are more resistant than WT cells in response to mitoxantrone, cisplatin, and oxaliplatin, similarly susceptible to CytD and only partially resistant to Noco, paclitaxel, docetaxel, and vinblastine (Supplementary Fig. S1C). Altogether, these results indicate that deletion of Bax/Bak (or BAX/BAK) confers a variable degree of resistance to anticancer drugs, in line with previously published studies [32, 54].

**Fig. 2:**
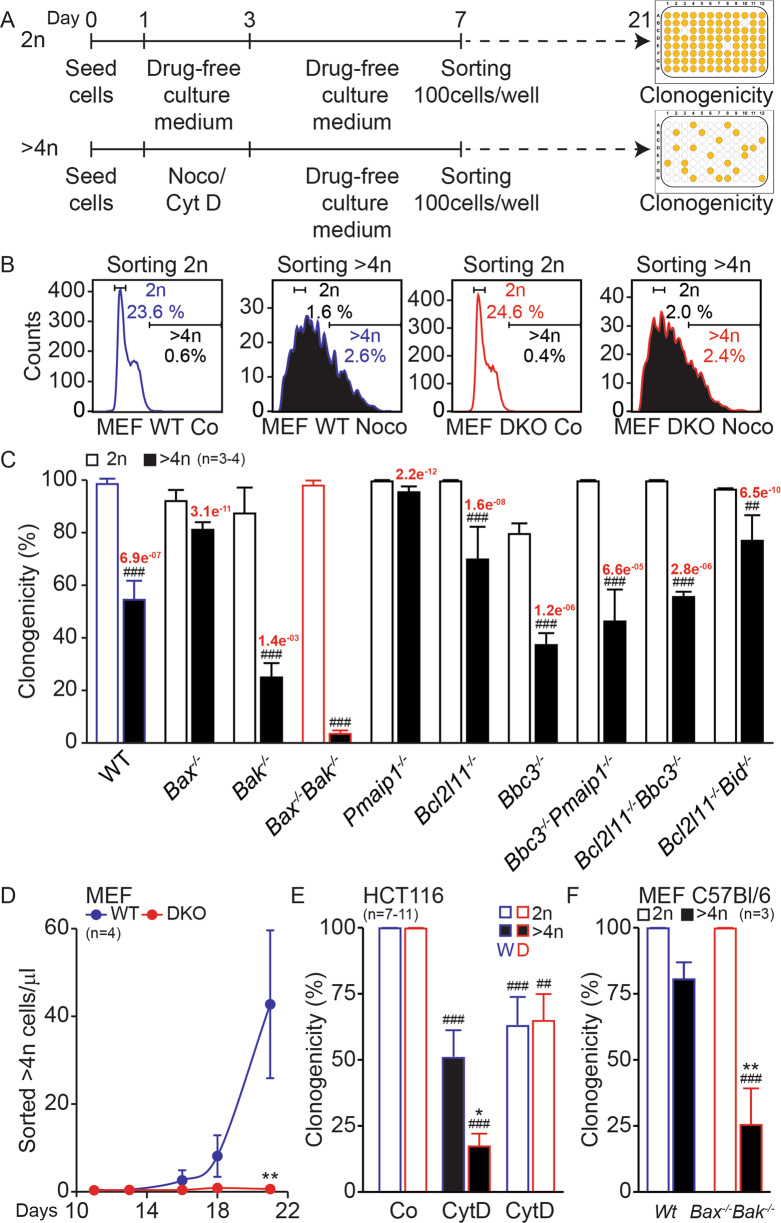
Knockout of Bax and Bak compromises clonogenic potential of tetraploid cells. Mouse embryonic fibroblasts (MEFs) (**A**–**D**), human colon carcinoma HCT116 cells (**E**), or immortalized MEFs derived from C57Bl/6 mice (MEF C57Bl/6) (**F**) were seeded on day 0, cultured from day 1 to day 3 (48 h) in drug-free medium or treated with 100 nM Nocodazole (Noco) (**A**–**D** and **F**) or 1.2 μM cytochalasin D (CytD) (**A**, **E**), then washed and kept in a drug-free medium for 4 more days, and finally sorted on day 7 (100 cells per well). Diploid (2*n*) cells were derived from cells grown in a drug-free medium (**A**–**C**, **E**, **F**) or from cells treated with CytD (**E**), whereas polyploid (>4*n*) cells were derived from cells treated with Noco (**A**–**D** and **F**) or CytD (**E**). The cell cycle and the DNA content gates were established by cell staining with 10 μM Hoechst 33342 for 1 h (**B**). Clonogenicity of MEF (**C**), HCT116 (**E**), or MEF C57Bl/6 (**F**) cells deficient for one or two pro-apoptotic proteins was quantified at day 21 as percentage of wells in which there was cell proliferation. Alternatively, >4*n* wild type (WT) and *Bax*^*−/−*^*Bak*^*−/−*^ (DKO) MEFs sorted on day 7 were subjected for cell proliferation assay at different time points between days 7 and 21 (**D**). Bars with blue contours represent WT cells and bars with red contours represent Bax/Bak DKO cells. White columns represent clonogenicity of 2*n* cells and black columns represent clonogenicity of >4*n* cells. Error bars indicate SEM. Data were compared by R software using standard linear model inference, “lm” function. >4*n* Samples were compared to 2*n* samples for each cell line (##*p* < 0.01, ###*p* < 0.001 vs. 2n). Clonogenicity of >4*n* cells was compared between all MEFs deficient for different pro-apoptotic proteins vs. DKO cells (*p*-values in red, **C**); or between DKO (“D”) and WT (“W”) cells (**E**, **F**) (*p < 0.05, **p < 0.01). Cell proliferation of >4*n* DKO (red) vs. >4*n* WT (blue) MEFs (**D**) was compared (**p < 0.01).

Intrigued by the fact that Bax/Bak (or BAX/BAK) deficiency confers relatively poor protection against cell cycle-perturbing agents (such as microtubular and spindle checkpoint inhibitors), we investigated the effects of Noco, which is a reversible microtubular inhibitor that can be washed out so that cells resume proliferation [55, 56]. WT and Bax/Bak MEFs responded similarly by an accumulation of tetraploid cells to transient exposure to Noco (Fig. 2A, B). Surprisingly, purified tetraploid Bax/Bak MEFs were unable to resume proliferation and hence were poorly clonogenic (<5% cells), whereas ~50% of WT MEFs were able to do so and formed macroscopic clones upon cytofluorometric sorting and culture in 96-well plates (Fig. 2A, C). We performed an extensive comparison of different MEF genotypes, finding that the single knockout of BAX had a minor effect, but that of BAK alone compromised clonogenic potential to about half of that of WT cells. However, the strongest phenotype was seen for the Bax/Bak DKO MEFs, whereas other DKOs (for instance of *Bbc3* and *Pmaip1*, *Bcl2l11*, and *Bbc3*, *Bcl2l11*, and *Bid*) had no such effects (Fig. 2C). Of note, DKO cells expressed close-to-normal levels (with variations up to 40%) of other genes from the BCL2 family (Supplementary Fig. S2).

Hence, the loss of clonogenicity of tetraploid cells is a feature of Bax/Bak DKO MEFs, which fail to resume proliferation after Noco washout (Fig. 2D). Tetraploid BAX/BAK DKO HCT116 cells also lost much of their clonogenic potential after CytD treatment as compared to tetraploid WT cells (Fig. 2E), confirming the data obtained with MEF. As an additional control, we took advantage of another source of WT and Bax/Bak DKO MEFs (which were derived from *Bax/Bak* KO mice), replicating the defective clonogenicity of tetraploid Bax/Bak DKO cells.

### Senescence of tetraploid Bax/Bak DKO cells

Next, we attempted to understand the reasons why Bax/Bak DKO cells lose their clonogenic survival upon tetraploidization. Noco undistinguishably induced ER stress in WT and Bax/Bak (or BAX/BAK) DKO cells, as indicated by the phosphorylation of eukaryotic initiation factor 2α and the nuclear translocation of the ER stress-linked transcription factors ATF6 and XBP1. These ER stress markers were determined by multicolor immunofluorescence staining and image cytometry, comparing WT and Bax/Bak DKO MEFs (Supplementary Fig. S3) or WT and BAX/BAK DKO HCT116 cells (Supplementary Fig. S4). Similarly, WT and Bax/Bak DKO MEFs produced similarly elevated levels of reactive oxygen species (ROS) in response to Noco, as measured with the ROS sensor Oxidative Stress Detection Reagent (Supplementary Fig. S5A) and contained similar levels of the autophagy markers LC3-II and SQSTM1 (also known as p62). Although an increase in LC3 lipidation is observed, autophagic flux is blocked after Noco treatmen, as, in neither line, lipidation is accompanied by effective degradation of SQSTM1 (Supplementary Fig. S5B). Finally, WT and Bax/Bak DKO MEFs manifested a similar increase in Noco-induced autophagosomes detectable by transmission electron microcopy (Supplementary Fig. S5C).

**Fig. 3:**
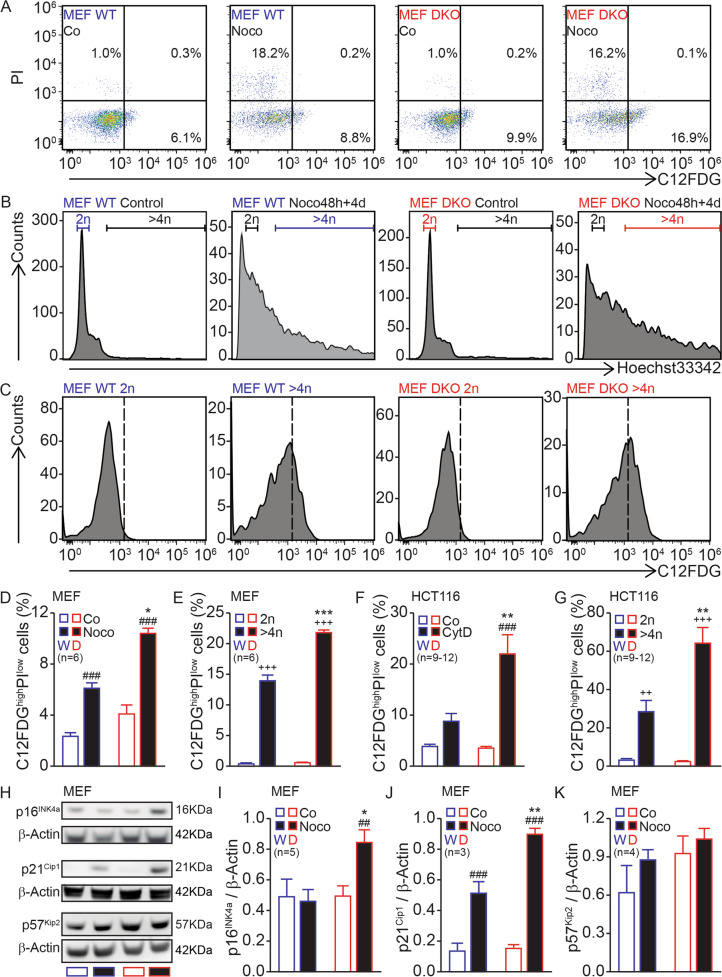
Bax/Bak DKO cells undergo senescence after induction of tetraploidy. Mouse embryonic fibroblasts (MEFs) (**A**–**E**, **H**–**K**) or human colon carcinoma HCT116 cells (**F**, **G**) were seeded, treated or not with 100 nM Nocodazole (Noco) or 1.2 μM cytochalasin D (CytD) for 48 h, then washed and kept in a drug-free medium for 4 days. Then, cells were stained with the vital dye propidium iodide (PI), the β-galactosidase substrate, C_12_FDG, and the fluorescent stain for DNA, Hoechst 33342. Live cells (**A**) were subjected to DNA content analysis (**B**). C12FDG was analyzed both in the total population (**D**, **F**) and according to ploidy (2*n* or >4*n*) (**C**, **E**, **G**). Alternatively, p16^INK4a^ (**H**, **I**), p21^Cip1^ (**H**, **J**), and p57^Kip2^ (**H**, **K**) in MEFs were determined by immunoblotting. Bars with blue contours represent WT (“W”) cells and bars with red contours represent Bax/Bak DKO (“D”) cells. White columns represent control or 2*n* cells and black columns represent treated or >4*n* cells. Error bars indicate SEM. Data were compared by R software using standard linear model inference, “lm” function. ##*p* < 0.01, ###*p* < 0.001 treatment vs. control. ++*p* < 0.01, +++*p* < 0.001 > 4*n* vs. 2*n*. *p < 0.05, **p < 0.01, **p < 0.001 for treatment effect, DKO vs. WT.

Next, we performed triple staining with the vital dye PI, the cell-permeable chromatin stain Hoechst 33342, and the β-galactose substrate C12FDG, a senescence marker [57]. Noco treatment led to a similar minor loss of viability among WT and Bax/Bak DKO MEFs. However, the percentage of C12FDG^high^ cells was higher among viable Bax/Bak DKO MEFs than WT controls (Fig. 3A, B). These C12FDG^high^ cells were especially elevated among hyperploid (>4*n*) cells (Fig. 3C), indicating that tetraploid Bax/Bak DKO cells are particularly prone to senescence (Fig. 3D, E). This result was replicated in HCT116 cells subjected to experimental tetraploidization, indicating that the lack of BAX/BAK predisposes to senescence (Fig. 3F, G). Moreover, Noco-induced higher levels of CDK inhibitor 2A protein p16^INK4a^ and CDK inhibitor 1A protein p21^Cip1^ in Bax/Bak DKO compared to WT MEFs, but not CDK inhibitor 1C protein p57^Kip2^, as determined by immunoblotting (Fig. 3H–K). In conclusion, it appears that the loss of Bax/Bak (or BAX/BAK) causes tetraploid cells to undergo a higher degree of senescence, explaining their loss of clonogenic potential.

### Aberrant Ca^2+^ signaling in tetraploid Bax/Bak DKO cells

**Fig. 4:**
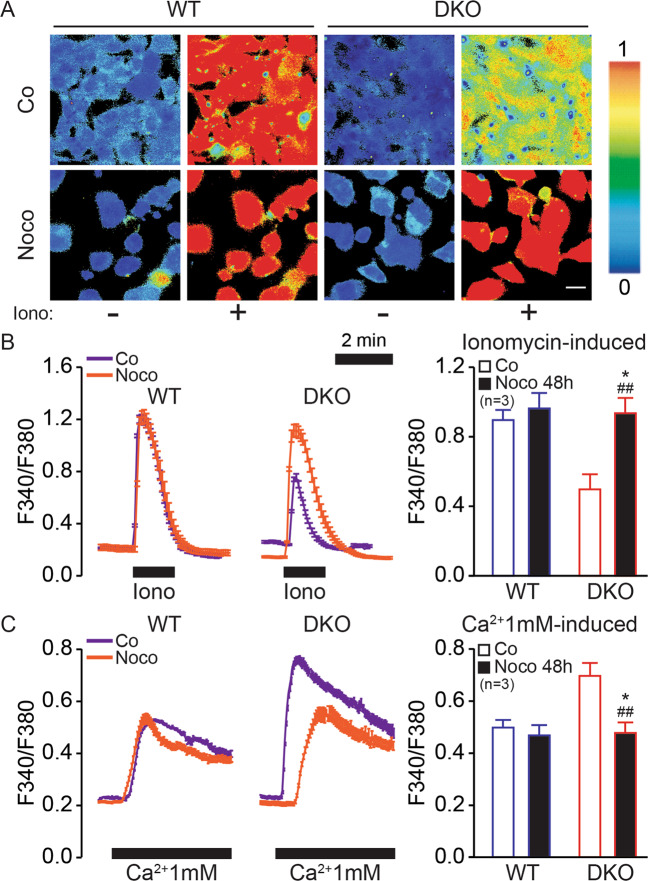
Bax/Bak DKO cells show altered Ca2+ fluxes. WT and DKO mouse embryonic fibroblasts (MEFs) were seeded and treated or not with 100 nM Nocodazole (Noco) for 48 h. Then, cells were MEFs loaded with 4 µM Fura2/AM and subjected to fluorescence imaging of cytosolic Ca^2+^ (**A**). Ca^2+^ store content (**A**, **B**) was evaluated by perfusing cells with 100 nM ionomycin (Iono) for 1 min in Ca^2+^-free medium. Store-operated Ca^2+^ entry (SOCE) (**C**) was evaluated by perfusing cells with 10 μM cyclopiazonic acid for 10 min or 1 µM thapsigargin for 10 min in Ca^2+^-depleted medium. Then, cells were perfused with 1 mM Ca^2+^-containing medium. **B**, **C** On the left are shown [Ca^2+^]_cyt_ traces of control (Co) cells (purple) and treated (Noco) cells (orange), and on the right the quantification. Bars with blue contours represent WT cells and bars with red contours represent Bax/Bak DKO cells. White columns represent control cells and black columns represent treated cells. Error bars indicate SEM. Data were compared by R software using standard linear model inference, “lm” function. ##*p* < 0.01 treatment vs. control. *p < 0.05, for treatment effect, DKO vs. WT. Scale bar, 10 μm.

**Fig. 5:**
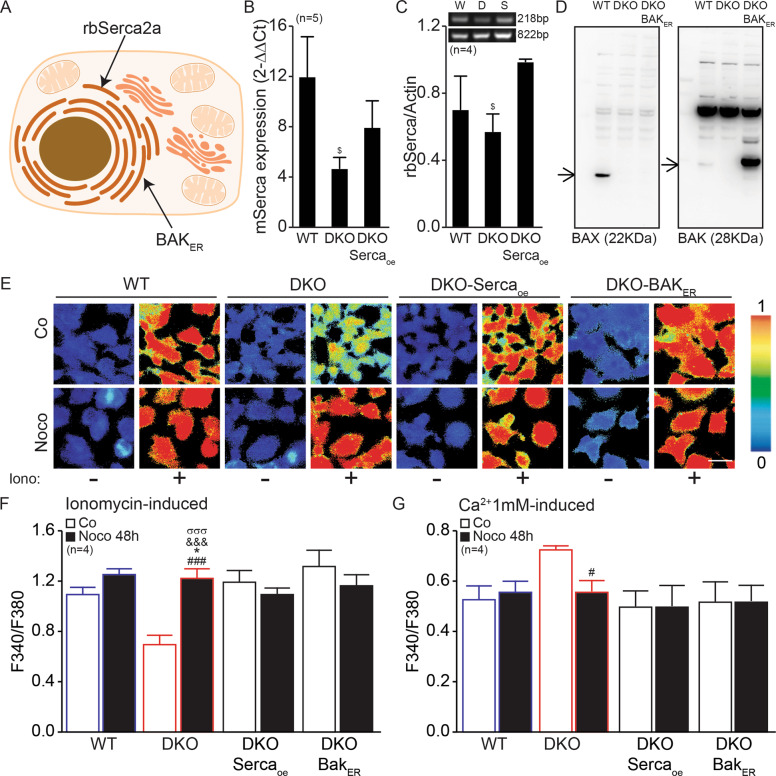
Modifications at the ER level reestablish Ca2+ fluxes in Bax/Bak DKO cells. WT, DKO, and DKO mouse embryonic fibroblasts (MEFs) stably transfected (**A**) with rabbit SERCA2a (DKO with Serca2a-overexpressed, DKO-Serca_oe_) (**B**, **C**), or an ER-targeted version of Bak (DKO-Bak_ER_) (**B**, **D**) were seeded and treated or not with 100 nM Nocodazole (Noco) for 48 h. Then, cells were MEFs were loaded with 4 µM Fura2/AM and subjected to fluorescence imaging of cytosolic Ca^2+^ (**E**). Ca^2+^ store content (**F**) was evaluated by perfusing cells with 100 nM ionomycin (Iono) for 1 min. Store-operated Ca^2+^ entry (SOCE) (**G**) was evaluated by perfusing cells with 10 μM cyclopiazonic acid for 10 min or 1 µM thapsigargin for 10 min in Ca^2+^-depleted medium. Then, cells were perfused with 1 mM Ca^2+^-containing medium. Bars with blue contours represent WT cells and bars with red contours represent Bax/Bak DKO cells. White columns represent control cells and black columns represent treated cells. Error bars indicate SEM. Data were compared by R software using standard linear model inference, “lm” function. $*p* < 0.05, DKO vs. WT in **B** or vs. DKO-Serca2a_oe_ in **C**. #*p* < 0.05, ###*p* < 0.001 treatment vs. control. *p < 0.05, for treatment effect, DKO vs. WT. &&&*p* < 0.001 for treatment effect, DKO vs. DKO-Serca_oe_. σσσ*p* < 0.001 for treatment effect, DKO vs. DKO-BAK_ER_. Scale bar, 10 μm.

BCL2 family proteins including BAX and BAK have been suspected to affect calcium (Ca^2+^) signaling, as determined by cell-free experiment and measurement of Ca^2+^ fluxes in stressed cells [13, 20, 22]. Bax/Bak DKO MEFs manifested a reduced ionomycin-induced release of Ca^2+^ from the ER into the cytosol at the basal level compared to WT MEFs, as determined with the ratiometric probe Fura-2AM and fluorescence microscopy (Fig. 4A, B). Of note, tetraploidization did not affect ER Ca^2+^ stores in WT cells, but did increase such stores in Bax/Bak DKO MEFs (Fig. 4A, B). Moreover, DKO cells manifested a higher SOCE than WT controls and this increase was reduced by Noco (Fig. 4A, C). Interestingly, DKO cells manifested an underexpression of endogenous Serca2a (official name Atp2a2) and overexpression of inositol 1,4,5-Trisphosphate Receptor Type 2 and 3 (Itpr2 and Itpr3), which might explain the effect on ER Ca^2+^ stores. Moreover, DKO cells overexpress stromal interaction molecule 1, which is involved in SOCE (Supplementary Fig. S6). Considering that restoration of [Ca^2+^]_ER_ in DKO cells with reintroduction of Serca2a and Bak into the ER has previously been described [22, 23], we wondered whether they would also play a role in restoring Ca^2+^ signaling in tetraploid Bax/Bak DKO cells. We took advantage of DKO cells transfected with rbSerca2a or an ER-targeted version of Bak (Bak_ER_) (Fig. 5A–D) and compared Ca^2+^ fluxes in untreated vs. Noco-treated WT, DKO cells, and DKO cells expressing rbSerca2a or Bak_ER_. Of note, the modulation of Ca^2+^ fluxes by the DKO (a decrease of ionomycin-induced cytosolic Ca^2+^ in untreated cells and an increase of store-operated Ca^2+^ in Noco-treated cells) was lost upon introduction of rbSerca2a or Bak_ER_ into these DKO cells (Fig. 5E–G).

### Reversion of tetraploid Bax/Bak DKO senescence by ER-targeted Bak

**Fig. 6:**
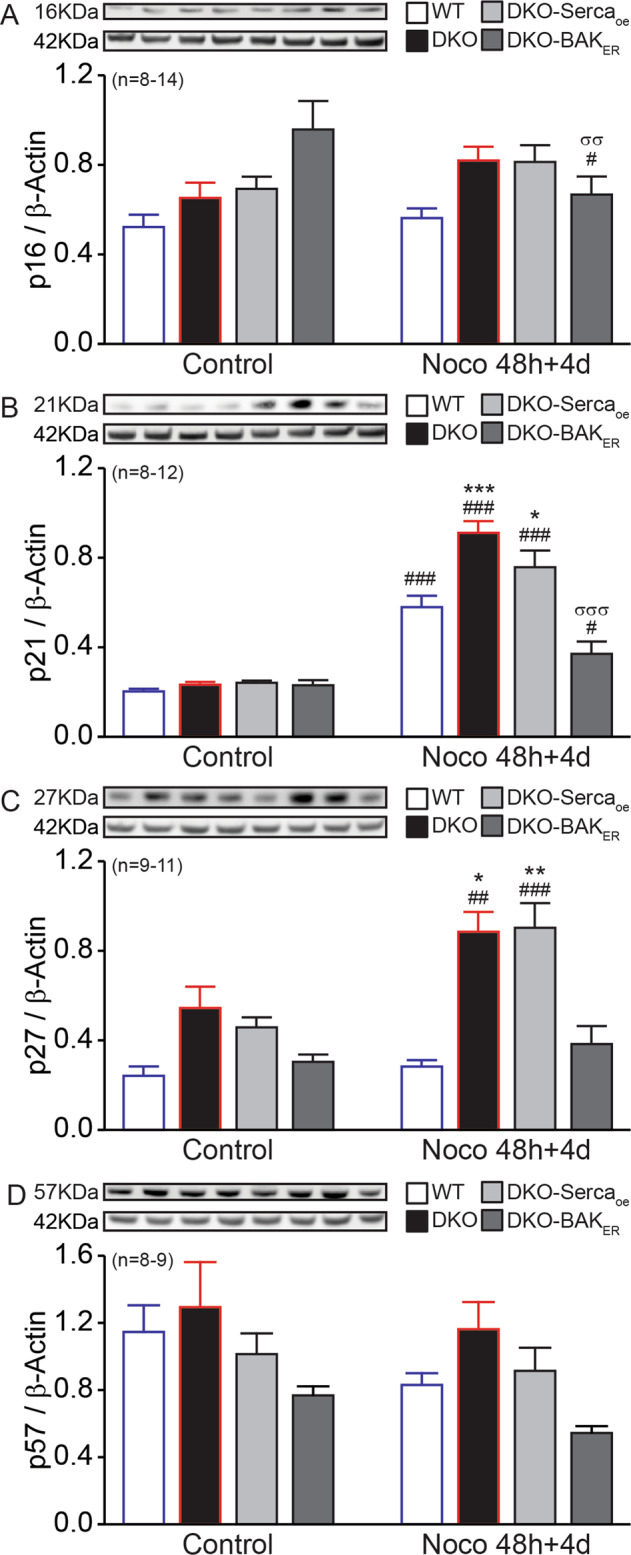
ER-targeted Bak reverses senescence of tetraploid Bax/Bak DKO. WT, DKO, and DKO with Serca2a-overexpressed (DKO-Serca_oe_), or DKO expressing an ER-targeted version of Bak (DKO-Bak_ER_) mouse embryonic fibroblasts (MEFs) were seeded, treated, or not with 100 nM Nocodazole (Noco) for 48 h, then washed and kept in a drug-free medium for 4 days. Attached cells were collected and p16^INK4a^ (**A**), p21^Cip1^ (**B**), p27^Kip1^ (**C**), and p57^Kip2^ (**D**) were determined by immunoblotting. Inserts show representative experiments. Densitometry data shown as mean value ± SEM are depicted. Bars with blue contours represent WT cells and bars with red contours represent Bax/Bak DKO cells. Data were compared by R software using standard linear model inference, “lm” function. #*p* < 0.05, ##*p* < 0.01, ###*p* < 0.001 treatment vs. control. *p < 0.05, **p < 0.01, ***p < 0.001, for treatment effect, DKO, or DKO-Serca_oe_ vs. WT. σσ*p* < 0.01, σσσ*p* < 0.001, for treatment effect, DKO vs. DKO-BAK_ER_.

The aforementioned results suggest that the Bax/Bak DKO and tetraploidization in Bax/Bak DKO cells affect Ca^2+^ fluxes, yet provide no prove that such alterations account for the senescence of tetraploid Bax/Bak DKO cells. We therefore measured markers of senescence in untreated vs. Noco-exposed WT, DKO MEFs, and DKO cells expressing rbSerca2a or Bak_ER_. Importantly, introduction of Bak_ER_ reduced the Noco-induced expression of the senescence markers p21^Cip1^ and cyclin-dependent kinase inhibitor 1B p27^Kip1^ to WT levels (Fig. 6). Therefore, the exclusive presence of Bak in the ER plays a key role in senescence induced by tetraploidizing agents.

**Fig. 7:**
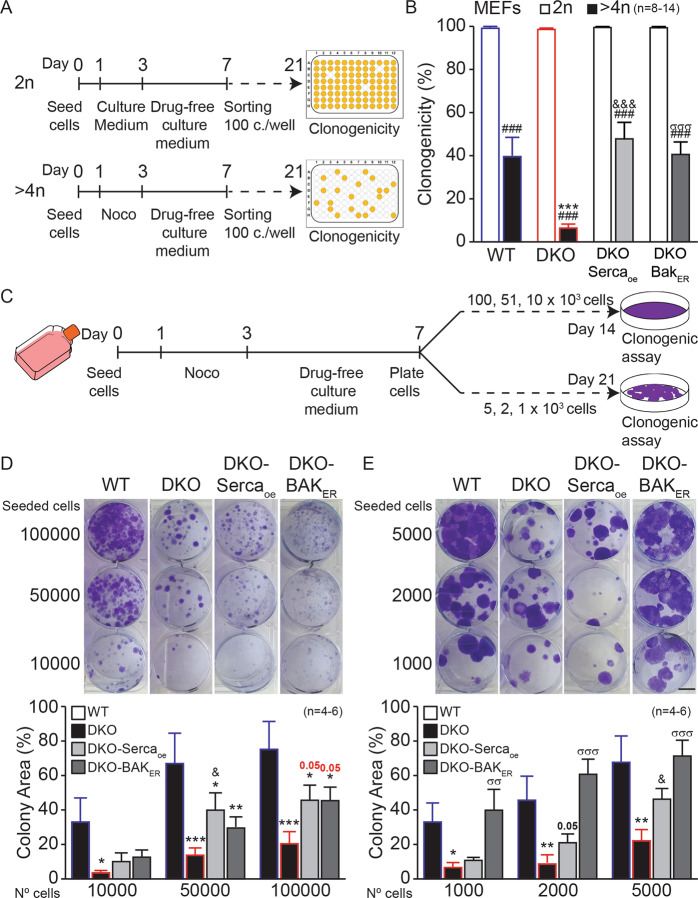
ER-targeted Bak SERCA2a restore the clonogenic potential of tetraploid Bax/Bak DKO cells. WT, DKO, and DKO with Serca2a-overexpressed (DKO-Serca_oe_), or DKO expressing an ER-targeted version of Bak (DKO-Bak_ER_) mouse embryonic fibroblasts (MEFs) were seeded, treated or not with 100 nM Nocodazole (Noco) for 48 h, then washed and kept in a drug-free medium for 4 days, and finally sorted (100 cells per well) on day 7. Diploid (2*n*) cells were derived from cells grown in a drug-free medium, whereas polyploid (>4*n*) cells were derived from cells treated with Noco (**A**). Clonogenicity of different MEFs was quantified at day 21 as percentage of wells in which there was cell proliferation (**B**). Alternatively, WT, DKO, DKO-Serca_oe_, or DKO-Bak_ER_ MEFs were seeded, treated with 100 nM Nocodazole (Noco) for 48 h, then washed and seeded again in a six-well plate at different cell concentrations. After 14 or 21 days, colonies were stained with crystal violet (**C**). Pictures were taken and colony area analyzed (**D**, **E**). Pictures show a representative experiment. Scale bar = 1 cm. Data shown as mean value ± SEM. White bars represent control cells and black or gray bars represent treated cells. Bars with blue contours represent WT cells, bars with red contours represent Bax/Bak DKO cells, light gray bars represent treated DKO-Serca_oe_ cells, and dark gray bars represent DKO-Bak_ER_ cells. Data in **B** were compared by R software using standard linear model inference, “lm” function. Data in **D** and **E** were compared by R software using linear mixed-effects model, “lme” function. ###*p* < 0.001 treatment vs. control. *p*-Values in black, *p < 0.05, ***p < 0.001, for treatment effect, DKO, DKO-Serca_oe_, or DKO-Bak_ER_ vs. WT. *p*-Values in red, &&&*p* < 0_._001 for treatment effect, DKO vs. DKO-Serca_oe_. *p*-Values in red, σσσ*p* < 0.001 for treatment effect, DKO vs. DKO-BAK_ER_.

At the functional level, both Bak_ER_ and SERCA2a were able to restore the clonogenic potential of tetraploid Bax/Bak DKO cells, as determined by two distinct experimental approaches. In the first protocol, WT, DKO MEFs, and DKO MEFs expressing rbSerca2a or Bak_ER_ were transiently cultured in Noco, washed, re-cultured without Noco, stained with Hoechst 33324, subjected to cytofluorometric purification of tetraploid cells, and seeded in 96-well plates (100 cells per well). Using this assay, we found that tetraploid DKO cells expressing rbSerca2a or Bak_ER_ recovered a normal clonogenic potential comparable to WT MEFs (Fig. 7A, B). As an alternative approach that does not involve Hoechst 33324 staining, cells were treated for 48 h with Noco, washed, allowed to rest for 4 days, and cultured in six-well plates for 2–3 weeks, followed by visualization of clones by crystal violet staining (Fig. 7C). Of note, only Bax/Bak DKO cells manifested a reduction in clonogenic potential as compared to WT controls. This phenotype was completely lost upon introduction of Bak_ER_ into the cells and was attenuated by SERCA2a as well (Fig. 7D, E).

We conclude that Ca^2+^ fluxes at the level of ER are indeed important to explain the propensity of Bax/Bak DKO cells to undergo senescence upon their tetraploidization.

## Discussion

The DKO of BAX and BAK, as well as the more recent TKO of BAX + BAK + BOK strongly inhibits the mitochondrial pathway of apoptosis. [33, 58]. Accordingly, MEFs from such DKO or TKO mice are largely resistant against a series of pro-apoptotic insults and similar results have been obtained in human DKO cells such as the HCT116 colon cancer cells used in this study. Here we report the unexpected finding that BAX and BAK are required for the expansion of tetraploid cells generated as a result of failed mitosis, supporting the idea that BAX and BAK are not only pro-apoptotic proteins but that they may also be required for assuring the fitness of cells in specific circumstances such as tetraploidization. Previous reports have demonstrated “moonlighting” functions for BCL2 family members that also regulate cell death-unrelated functions including autophagy, cellular senescence, inflammation, Ca^2+^ fluxes, bioenergetics, and redox homeostasis [59]. However, most of these “moonlighting” functions have been attributed to anti-apoptotic BCL2 family members and, to the best of our knowledge, this is the first report on a function of BAX/BAK that favors cellular fitness.

Although the exact pathway explaining how BAX/BAK is required for the proliferation of tetraploid cells remains to be elucidated, our present study highlights two elements in this pathway. First, the presence of BAX/BAK is required for tetraploid cells to suppress a permanent cell cycle arrest referred to as senescence. Following the exposure of cells to the reversible spindle poison Noco (which causes tetraploidization due to “mitotic slippage”) [55, 56], as well as its removal, BAX/BAK-deficient cells usually activate a senescence program, as indicated by the upregulation of several CDK inhibitors and the activation of senescence-associated β-galactosidase. It is well accepted that upregulation of p21 is not sufficient to maintain long-term senescent cell arrest. However, upregulation of p16 is able to maintain the senescent state over time [60]. This would explain why DKO cells cannot resume proliferation, whereas WT can do so. Second, compared to WT, the absence of BAX/BAK is tied to alterations in Ca^2+^ signaling with reduced ER Ca^2+^ stores and increased SOCE, yet restored by expressing SERCA2A or an ER-targeted version of BAK in the cells. Both these changes are lost after induction of tetraploidy in BAX/BAK DKO cells. ER-targeted BAK also restore the tetraploidy-associated senescence found in BAX/BAK DKO cells to BAX/BAK-sufficient control cell levels. Collectively, these findings suggest that BAX/BAK act on the ER to affect local Ca^2+^ fluxes, e.g., by downregulation of SERCA2A and upregulation of IP3R2 and IP3R3 in DKO cells, which then indirectly affect SOCE and senescence. Several models have shown that Ca^2+^ is involved in senescence. For example, IP3Rs [61, 62] and l-type Ca^2+^ channels appear to play a role in senescence in vascular smooth muscle cells [63] and β-cells from pancreatic islets [64]. Moreover, so-called BH3 mimetics, which are small molecules that activate MOMP (and also mediate other effects including the induction of autophagy) [65, 66] have been shown to eliminate senescent cells from aging tissues and consequently to mediate “senolysis” [67–69]. Molecular connections have been established between some members of the BCL2 family and senescence. However, the precise molecular links between ER Ca^2+^ storage, BAX/BAK, and senescence remain to be elucidated.

It is fascinating to consider that, depending on the exact genetic background, BAX/BAK-knockout mice are usually born at the expected frequency and then are variably affected by perinatal mortality. Such mice develop close-to-normally, with relatively minor phenotypes, but do not develop malignant disease as a major phenotype [39]. Genetic studies investigating the ploidy of cancer cells have led to the conclusion that at least 40% of solid cancers are the result of an often transient tetraploidization followed by the loss of excessive chromosomes to reestablish a close-to-diploid (but often aneuploid) karyotype [70]. This polyploidization/deploidization cycle may be considered as a major mechanism of genomic instability that favors oncogenesis and tumor progression [70], and is also under the control of the immune system (which tends to eliminate tetraploid or higher-order polyploid cells) [71, 72]. Although formal proof for this conjecture is elusive, it is tempting to speculate that BAX/BAK deficiency is not a major driver of oncogenesis, because it compromises the proliferative potential of tetraploid cells, hence obliterating one major path towards carcinogenesis. Following the same mental construction, the rarity of complete loss of BAX and BAK from cancer cells may be explained. Indeed, tumor cells have to navigate between Scylla and Charybdis, to avoid their death (which often occurs through apoptosis) and their senescence (which is another effective tumor suppressor mechanism). From the point of view of the cancer cell, the loss of BAX/BAK may be desirable for avoiding apoptosis but disastrous for preventing senescence induced by ploidy changes and perhaps other yet-to-be elucidated stressors.

Irrespective of the aforementioned gaps in our knowledge, uncertainties on mechanisms, and speculative issues, it appears clear that the maintenance of BAX/BAK expression may be advantageous for cells in specific circumstances, as illustrated here for tetraploidization.

## Supplementary information

Supplementary Information

Supplementary Figure 1

Supplementary Figure 2

Supplementary Figure 3

Supplementary Figure 4

Supplementary Figure 5

Supplementary Figure 6

Supplementary Table

Author Contribution Form

Reproducibility Checklist

## Data Availability

Data are available in ArrayExpress under the ID “E-MTAB-10997” and through the corresponding author on request.
