# Supplementary material for: Paradoxical implication of BAX/BAK in the persistence of tetraploid cells
Source: Cell Death Dis. 2021 Nov 1;12(11):1039. doi: 10.1038/s41419-021-04321-3 (PMC8560871; doi:10.1038/s41419-021-04321-3)
Supplement: Supplementary file 1 — Supplementary Information [file 41419_2021_4321_MOESM1_ESM.docx]

**Supplementary Information**

**Material and Methods**

**Antibodies.** EIF2S1(phospho S51) (ab32157), rabbit polyclonal antibodies against XBP1 (ab37152), ATF6 (ab37149) were purchased from Abcam (Cambridge, UK). LC3B (#2775) was purchased from Cell Signaling, and SQSTM1 (H00008878-M01) monoclonal antibody from Abnova.

**Cytofluorometry.** *Cell death.* HCT116 cells were seeded in 12-wells plates. Next day, cells were treated with the following pharmacological agents: mitoxantrone 1 μM, CDDP 10 μM, OXA 50 μM, Noco 100nM, PCT 300 nM, docetaxel (100 nM), vinblastine (4 μM), or cytochalasin D (Cyt D, 1.2 μM) from MP Biomedicals (Santa Ana, CA, USA). After 48 h of treatment, apoptosis was measured in unfixed cells co-stained with 40 nM 3,3'dihexiloxalocarbocyanine iodide (DiOC_6_(3)) (Life Technologies Carlsbad, CA, Estados Unidos) to quantify mitochondrial transmembrane potential (ΔΨm) plus 1 μg/mL propidium iodide (PI) (Life Technologies) to identify plasma membrane breakdown.

*Reactive oxygen species (ROS)*. Cells were seeded in 12-wells plates. Next day, cells were treated with Noco 100 nM. After 48 h of treatment or 48 h of treatment + 4 days in drug-free culture medium, ROS production in live cells was labeled with oxidative stress detection reagent included into ROS/Superoxide Detection Assay Kit (Cell-based) (ab139476) (Abcam) following manufacturer’s instructions.

Cytofluorometric determinations were performed by means of an Attune® LSRII Fortessa flow cytometer (BD Biosciences) (San Jose, CA, USA). Data analyses were carried out by using the FlowJo software, upon gating on the events characterized by normal forward scatter and side scatter values.

**Imaging flow cytometry.** *Unfolded protein response (UPR).* Cells were seeded into 75 or 175 cm^2^ flasks for control and treated conditions, respectively. After 24 h, cells were treated with Noco 100 nM or cytochalasin D 1.2 μM for 48 h. Attached cells were either collected or cultured in drug-free medium for 4 d more and collected. 1× 10^6^ cells were fixed with freeze 70 % EtOH and stored at -20 ºC at least overnight. Then, cells were washed with phosphate-buffered saline (PBS) and permeabilized with 0.3 % Triton X-100 (Sigma-Aldrich) for 10 min. After, cells were washed again with PBS and unspecific antibody binding was reduced with blocking buffer (5 % bovine serum albumin (BSA) (w/v) in PBS) for 30 min followed by a consecutive multiple staining. First, cells were labeled with P-eIF2α primary antibody (1:50 in blocking buffer) for 1 h at room temperature (RT), washed with PBS and stained with Alexa fluor (AF) 568 secondary antibody (diluted 1:500) 30 min at RT. After wash with PBS, cells were labeled with ATF6 primary antibody (1:100) 1 h at RT, washed with PBS and stained with AF657 (1:500). Finally, cells were labeled with XBP1 primary antibody (1:100) 1h at RT, washed with PBS and stained with AF488 secondary antibody (1:500) + DAPI (1:1000, Life Technologies). Multispectral imaging flow cytometry was performed on an AMNIS ImageStream X Mark II equipped with 375-, 488-, 561-, and 642-nm lasers using the 60x magnification lens. At least 10,000 events were acquired for each sample. The analysis was done with IDEAS software version 6.2.187.0. Only focused events were included in the analysis, using the gradient RMS feature of bright field images. Singlets were then gated on aspect ratio *vs* area of bright field. A compensation matrix was calculated using single color fluorescent control files. This matrix was applied to each file. A morphology mask was applied on the DAPI-stained part of the cells to identify the nuclear area, whereas the cytoplasm was defined as the extranuclear area delimited by the nuclear area and the outer limit of the cell defined by bright field. The intensity of staining was quantified within these 2 masks. DAPI staining was used for cell cycle and DNA content analysis and gating.

**Transmission electron microscopy**. Cells were seeded in 100 mm cell culture dishes. After 24h, cells were treated with Nocodazole 100 nM for 48h. Then, cells were fixed for 1 h at 4 °C in 2 % glutaraldehyde in 0.1 M Sörensen phosphate buffer (pH 7.3), post-fixed for 1 h in aqueous 2 % osmium tetroxide, bloc-stained in 2 % uranyl acetate in 30 % methanol, dehydrated, and embedded in Epon™ 812. Ultrathin sections (80 nm) were stained with uranyl acetate and lead citrate. Sections were examined with Tecnai 12 electron microscope (FEI, Eindhoven, The Netherlands). Digital images were taken with a SIS MegaviewIII CCD camera.

**Transcriptomic analysis.** Three samples issued from MEF WT and MEF DKO both control and treated with Noco 100 nM for 48h were given to the genomic platform of Gustave Roussy Institute to perform a whole genome expression array in the context of the Taxe d’Apprentissage TA P28_JUHU 2018. Bioinformatics data are extracted from Agilent SurePrint G3 Mouse GE 8x60K Microarray (028005 / G4858A). Probes with more than 15% missing values among samples were discarded. Remaining missing values were imputed using k-nearest neighbor (kNN). Probe intensities were transformed to log (base 2), then reduced to the gene level using their median value. Quantile normalization was applied to the matrix. Differential analysis has been performed with the limma R-package for RNA-sequencing and microarray studies (1). Data have been deposited at ArrayExpress, under the ID “E-MTAB-10997”.

**References**

1. Ritchie ME, Phipson B, Wu D, Hu Y, Law CW, Shi W, et al. limma powers differential expression analyses for RNA-sequencing and microarray studies. Nucleic Acids Res. 2015;43(7):e47.

**Legends to Supplementary Items**

**Supplementary Figure 1. Cells deficient for BAX and BAK (DKO) are susceptible to killing by tetraploidizing agents.** Mouse embryonic fibroblasts (MEFs, A) and human colon cancer cell line, HCT116 (B), were subjected to western blot to confirm Bax and Bak deficiency in double KO (DKO) cells compared to wild-type (WT) cells. Wild type (W) and DKO (D) HCT116 cells were cultured for 48 h in the absence (control, Co) or presence of 1 μM mitoxantrone (MTX), 10 μM *cis*-diaminodicloroplatino(II) (CDDP), 50 μM oxaliplatin (OXA), 100 nM nocodazole (Noco), 1.2 μM cytochalasin D (Cyt D), 300 nM paclitaxel (PCT), 100 nM docetaxel (DTX), or 4 μM vinblastine (VB). Then, cells were stained with the vital dye propidium iodide (PI) and the mitochondrial membrane potential (ΔΨ_m_) – sensitive dye DiOC_6_(3) to measure apoptotic cell death (C). Black columns represent the percentage of dead (PI^high^) cells and white columns, dying cells (DiOC_6_(3)^low^PI^low^). Error bars indicate SEM. Total columns (DiOC_6_(3)^low^PI^low^ + PI^high^) were compared by R software using standard linear model inference, ‘lm’ function. # *p* < 0.05, ### *p* < 0.001 treatment *vs* control. *** *p* < 0.001 treatment effect, DKO (“D”, in red) *vs* WT (“W”, in blue).

**Supplementary Figure 2. The absence of Bax and Bak modifies in a limited way the expression of other Bcl-2 family proteins.** Wild-type (WT) and Bax Bak double KO (DKO) mouse embryonic fibroblasts (MEFs) were cultured in the absence (control, Co) or presence of 100 nM nocodazole (Noco) for 48h. Samples were subjected to a whole genome expression array analysis. Boxplots represent probe-set expression for different Bcl-2 family proteins. Blue boxplots represent WT cells and red boxplots represent DKO cells. Error bars indicate SEM. *p* value annotation is based on differential analysis within limma R-package.

**Supplementary Figure 3. Both wild-type (WT) and Bax Bak double KO (DKO) mouse embryonic fibroblasts (MEFs) exhibit endoplasmic reticulum stress after tetraploidy induction.** WT and DKO MEFs were cultured for 48 h in the absence (control, Co) or presence of 100 nM nocodazole (Noco) for 48 h. Then, attached cells were either collected (Noco 48h) or cultured in drug-free medium for 4 d more and collected (Noco 48h + 4d). Samples were simultaneously stained against XBP1, P-eIF2α and ATF6 (A). The mean fluorescence intensity (MFI) of XBP1 in the nucleus (nuc_XBP1), MFI of P-eIF2α in the cytoplasm (cyt_ P-eIF2α) and MFI of ATF6 in the nucleus (nuc_ATF6) was analyzed in diploid (2n) and tetraploid (4n) cells in B, C and D, respectively. (A) illustrates pictures of representative cells. Scale bar = 7 µm. Columns represent MFI ± SEM in control (white), after 48 h of treatment (grey) or 48 h of treatment + 4 d cultured in drug-free medium (black). Columns with blue contours represent WT cells and columns with red contours represent Bax/Bak DKO cells. Data were compared by R software using standard linear model inference, ‘lm’ function. + *p* < 0.05, ++ *p* < 0.01, +++ *p* < 0.001 4n *vs* 2n.

**Supplementary Figure 4. Human colon cancer cell lines, HCT116, wild-type (WT) and double KO for BAX and BAK (DKO) exhibit similar endoplasmic reticulum stress after tetraploidy induction.** WT and DKO HCT116 cells were cultured for 48 h in the absence (control, Co) or presence of 100 nM cytochalasin D (CytD) for 48 h. Then, attached cells were either collected (Noco 48h) or cultured in drug-free medium for 4 d more and collected (Noco 48h + 4d). Samples were simultaneously stained against XBP1, P-eIF2α and ATF6 (A). The mean fluorescence intensity (MFI) of XBP1 in the nucleus (nuc_XBP1), MFI of P-eIF2α in the cytoplasm (cyt_ P-eIF2α) and MFI of ATF6 in the nucleus (nuc_ATF6) was analyzed in diploid (2n) and tetraploid (4n) cells in B, C and D, respectively. (A) illustrates pictures of representative cells. Scale bar = 7 µm. Columns represent MFI ± SEM in control (white), after 48 h of treatment (grey) or 48 h of treatment + 4 d cultured in drug-free medium (black). Columns with blue contours represent WT cells and columns with red contours represent Bax/Bak DKO cells. Data were compared by R software using standard linear model inference, ‘lm’ function. + *p* < 0.05, ++ *p* < 0.01, +++ *p* < 0.001 4n *vs* 2n; # *p* < 0.05, ## *p* < 0.01 treatment *vs* control.

**Supplementary Figure 5. Mouse embryonic fibroblasts, MEF,** **wild-type (WT) and double KO for BAX and BAK (DKO) exhibit similar levels of autophagy.** WT and DKO MEFs were cultured for 48 h in the absence (control, Co) or presence of 100 nM nocodazole (Noco) for 48 h. Then, attached cells were either collected (Noco 48h) or cultured in drug-free medium for 4 d more and collected (Noco 48h + 4d). Levels of reactive oxygen species (ROS) were measured by flow cytometry (A), pellets from these samples were used to analyze changes in the autophagy markers LC3-II and SQSTM1 by western blot (B), as well as nocodazole-induced autophagosomes were detected in glutaraldehyde-fixed cells by transmission electron microcopy (C). A western blot of a representative experiment is shown in B. In C, representative electron microscopy images in control and after treatment with nocodazole at 48h are shown at the top. The images on the bottom are a magnification of the image corresponding to the treatment with nocodazole. Red asterisks point the autophagosomes. ER, Endoplasmic reticulum; GA, Golgi apparatus; Mit, mitochondrion; Nuc, Nucleus. Scale bars, 1µm. Data were compared by R software using standard linear model inference, ‘lm’ function. # *p* < 0.05, ## *p* < 0.01, ### *p* < 0.001 treatment *vs* control.

**Supplementary Figure 6. Bax and Bak deficiency affects gene expression of proteins involved in calcium fluxes.** Wild-type (WT) and Bax Bak double KO (DKO) mouse embryonic fibroblasts (MEFs) were cultured in the absence (control, Co) or presence of 100 nM nocodazole (Noco) for 48h. Samples were subjected to a whole genome expression array analysis. Heatmap represents gene selection for proteins involved in calcium fluxes normalized by row.
