# Supplementary figures and images for: Paradoxical implication of BAX/BAK in the persistence of tetraploid cells
Source: Cell Death Dis. 2021 Nov 1;12(11):1039. doi: 10.1038/s41419-021-04321-3 (PMC8560871; doi:10.1038/s41419-021-04321-3)

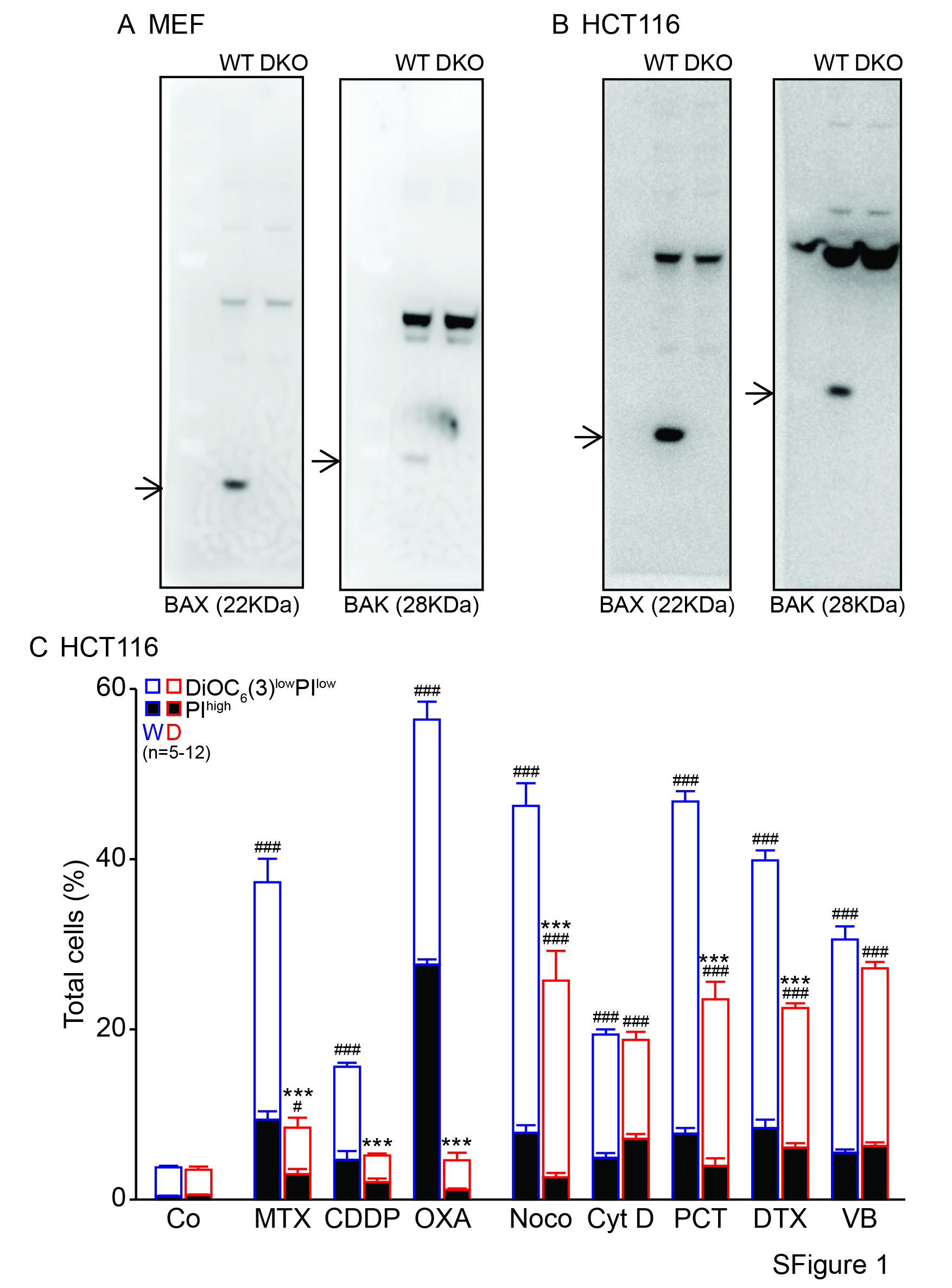

Supplement: Supplementary file 2 — Supplementary Figure 1 [file 41419_2021_4321_MOESM2_ESM.tif]

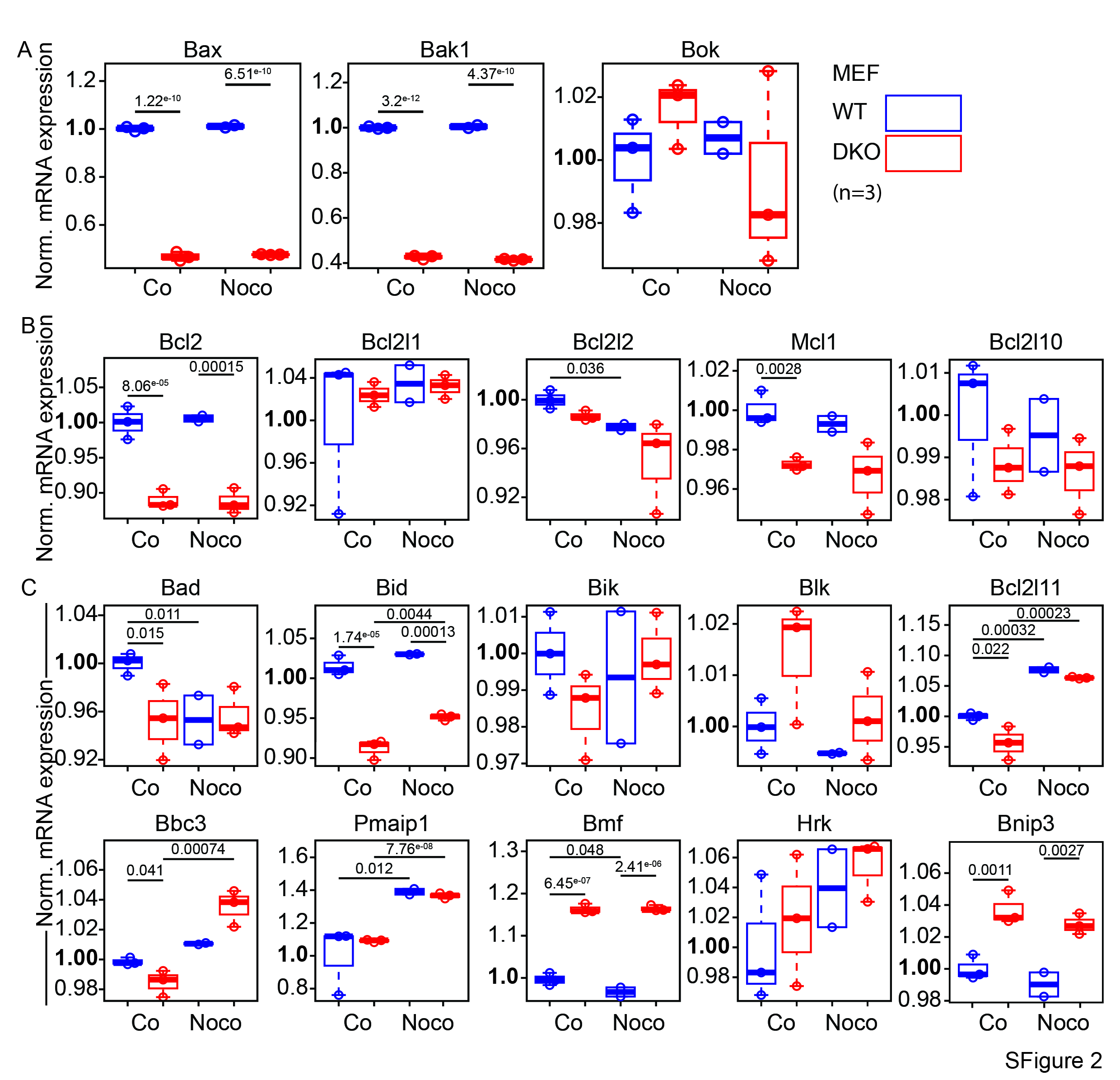

Supplement: Supplementary file 3 — Supplementary Figure 2 [file 41419_2021_4321_MOESM3_ESM.tif]

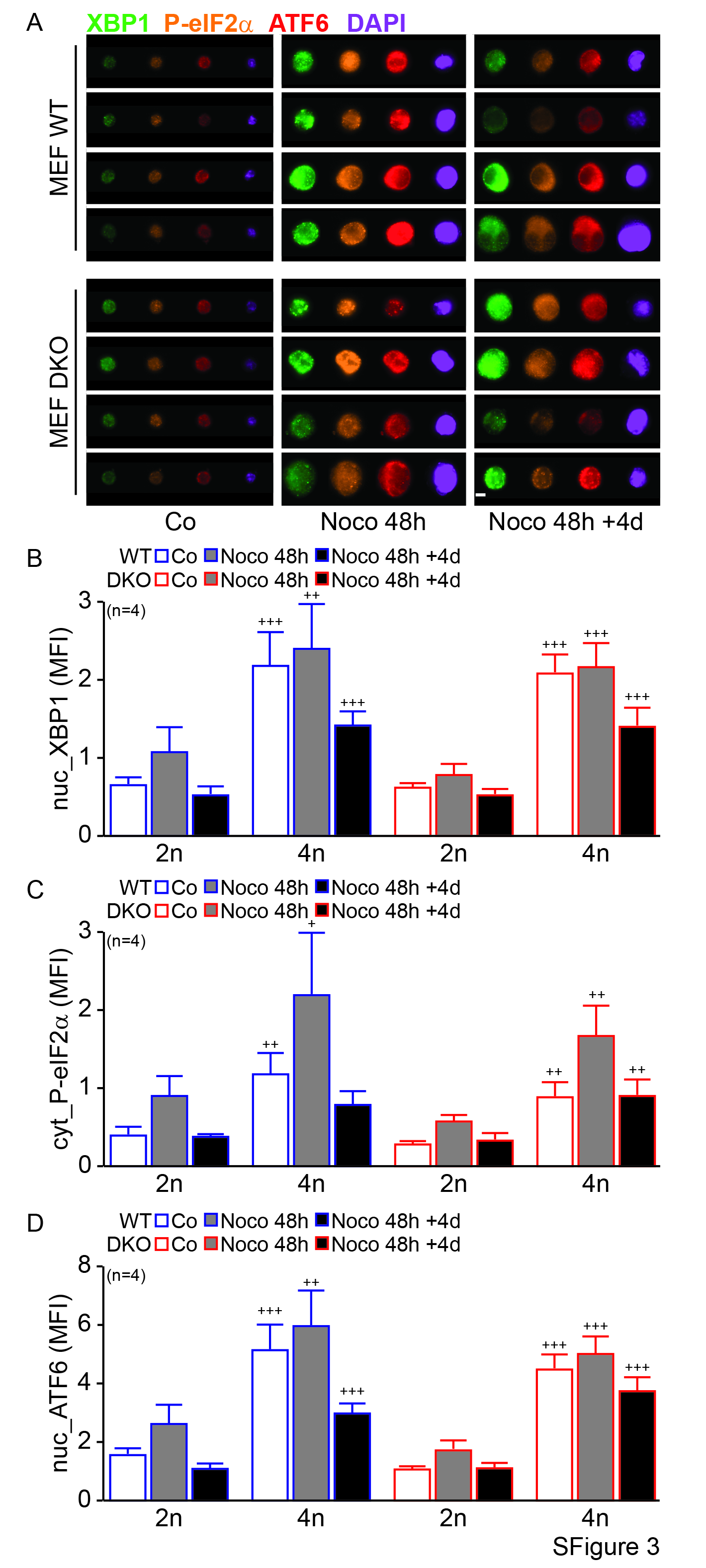

Supplement: Supplementary file 4 — Supplementary Figure 3 [file 41419_2021_4321_MOESM4_ESM.tif]

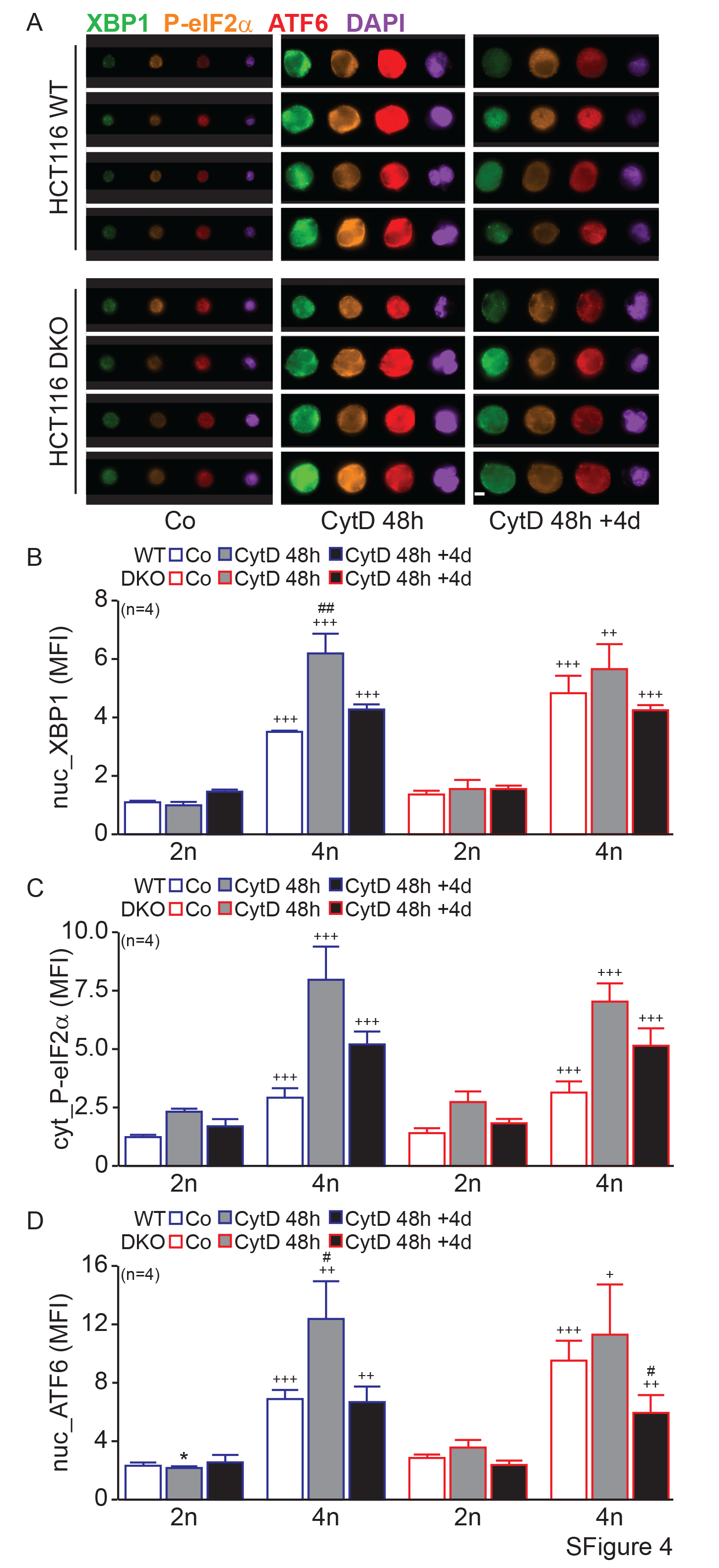

Supplement: Supplementary file 5 — Supplementary Figure 4 [file 41419_2021_4321_MOESM5_ESM.tif]

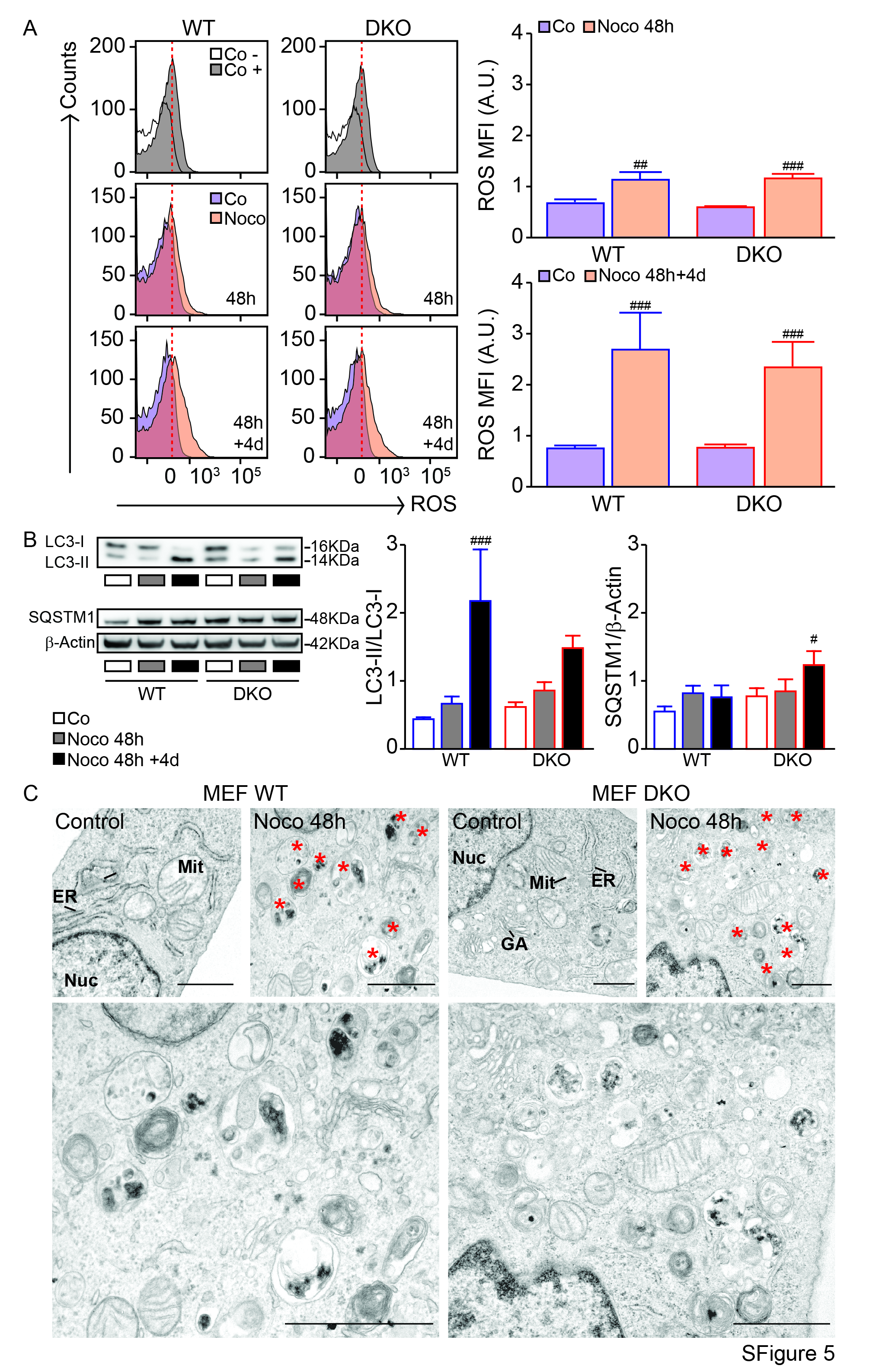

Supplement: Supplementary file 6 — Supplementary Figure 5 [file 41419_2021_4321_MOESM6_ESM.tif]

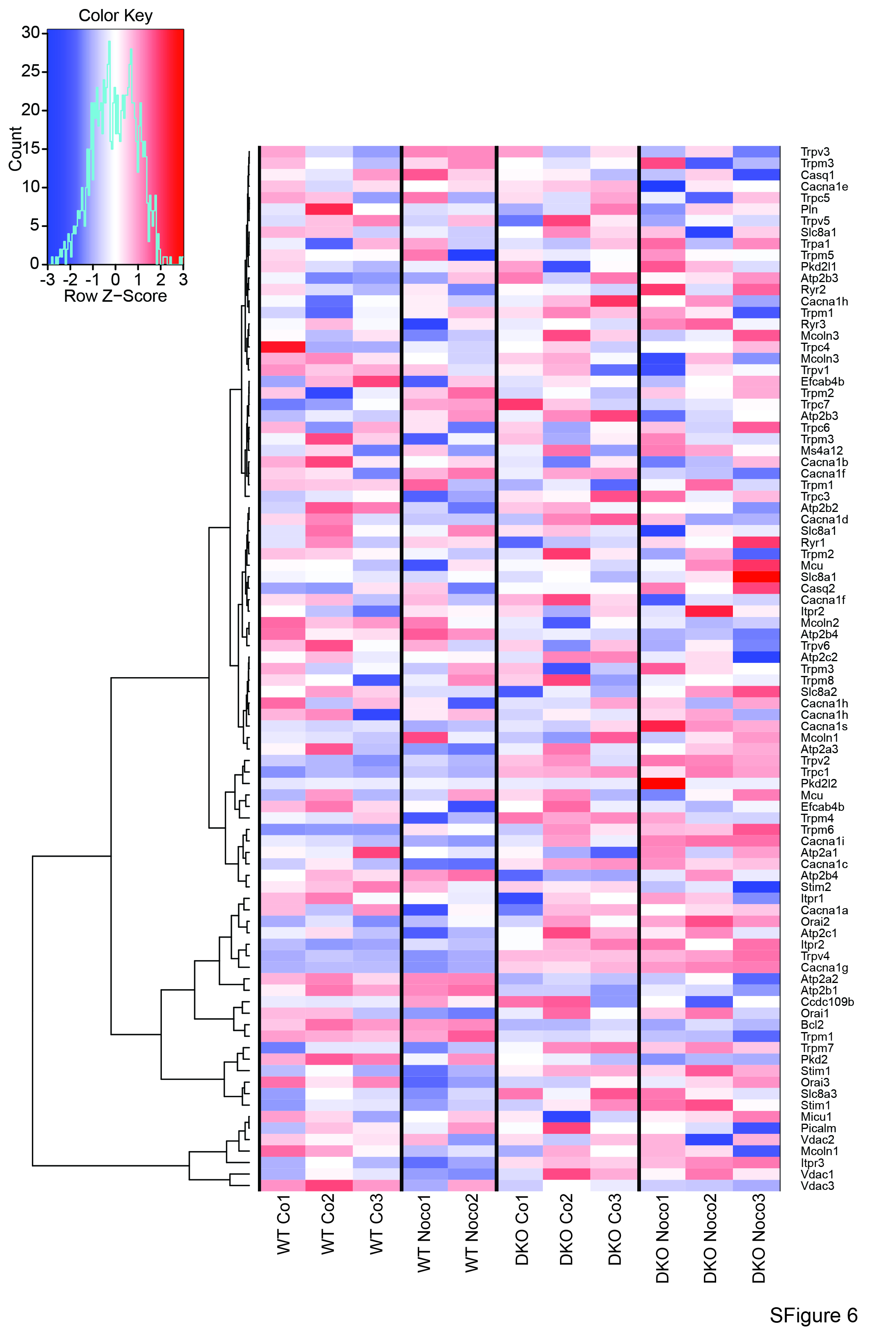

Supplement: Supplementary file 7 — Supplementary Figure 6 [file 41419_2021_4321_MOESM7_ESM.tif]
