# Supplementary material for: Paradoxical implication of BAX/BAK in the persistence of tetraploid cells
Source: Cell Death Dis. 2021 Nov 1;12(11):1039. doi: 10.1038/s41419-021-04321-3 (PMC8560871; doi:10.1038/s41419-021-04321-3)
Supplement: Supplementary file 8 — Supplementary Table [file 41419_2021_4321_MOESM8_ESM.pdf]

Table 1. R functions used in the statistical analysis of each of the figures.

Fig.	Transformation	Model	Variables	R function
**1B**	None	Linear	Mut(WT,DKO);Treat(C, CDDP 10uM,CDDP 20uM,CDDP 40uM)	lm(Y ~ Mut/Treat);lm(Y ~ Mut*Treat)
**1C**	None	Linear	Mut(WT,DKO);Treat(Co,Oxal_10uM,Oxal_25uM,Oxal_50uM)	lm(Y ~ Mut/Treat);lm(Y ~ Mut*Treat)
**1D**	None	Linear	Mut(WT,DKO);Treat(Co,Carb1uM,Carb100uM,Carb200uM)	lm(Y ~ Mut/Treat);lm(Y ~ Mut*Treat)
**1E**	None	Linear	Mut(WT,DKO);Treat(Co,NOCO50nM,NOCO100nM,NOCO200nM)	lm(Y ~ Mut/Treat);lm(Y ~ Mut*Treat)
**1F**	None	Linear	Mut(WT,DKO);Treat(Co,PCT150nM,PCT300nM,PCT600nM)	lm(Y ~ Mut/Treat);lm(Y ~ Mut*Treat)
**1G**	None	Linear	Mut(WT,DKO);Treat(Co,ROT1uM,ROT5uM,ROT10uM)	lm(Y ~ Mut/Treat);lm(Y ~ Mut*Treat)
**1H**	None	Linear	Mut(WT,DKO);Treat(Co,RO5uM,RO25uM,RO50uM)	lm(Y ~ Mut/Treat);lm(Y ~ Mut*Treat)
**1I**	None	Linear	Mut(WT,DKO);Treat(Co,AZD0.5uM,AZD5uM,AZD50uM)	lm(Y ~ Mut/Treat);lm(Y ~ Mut*Treat)
**1J**	None	Linear	Mut(WT,DKO);Treat(Co,M2I25uM,M2I50uM,M2I100uM)	lm(Y ~ Mut/Treat);lm(Y ~ Mut*Treat)
**2C**	None	Linear	Mut(Bak,Bax,BaxBak,Bim,BimBid,BimPuma,Noxa,Puma,PumaNoxa,WT);Chrom(>4n,2n)	lm(Y ~ Mut / Chrom);lm(Y ~ Chrom * Mut)
**2D**	None	Linear	Mut(WT,DKO);Day("11,13,16,18,21)	lm(Y ~ Mut/Day);lm(Y ~ Mut*Day)
**2E**	None	Linear	Mut(WT,DKO);TreatChrom(2nCo,4nCytD,2nCytD)	lm(Y ~ Mut/TreatChrom);lm(Y ~ TreatChrom*Mut)
**2F**	None	Linear	Mut(WT,BaxBak);Chrom(2n,4n)	lm(Y ~ Mut/Chrom);lm(Y ~ Chrom*Mut)
**3D**	None	Linear	Mut(WT,DKO);Treat(Co,Noco)	lm(Y ~ Mut/Treat);lm(Y ~ Mut*Treat)
**3E**	None	Linear	Mut(WT,DKO);Chrom(2n,4n)	lm(Y ~ Mut/Chrom);lm(Y ~ Mut*Chrom)
**3F**	None	Linear	Mut(WT,DKO);Treat(Co,CytD)	lm(Y ~ Mut/Treat);lm(Y ~ Mut*Treat)
**3G**	None	Linear	Mut(WT,DKO);Chrom(2n,4n)	lm(Y ~ Mut/Chrom);lm(Y ~ Mut*Chrom)
**3I**	None	Linear	Mut(WT,DKO);Treat(Co,Noco)	lm(Y ~ Mut/Treat);lm(Y ~ Mut*Treat)
**3J**	None	Linear	Mut(WT,DKO);Treat(Co,Noco)	lm(Y ~ Mut/Treat);lm(Y ~ Mut*Treat)
**3K**	None	Linear	Mut(WT,DKO);Treat(Co,Noco)	lm(Y ~ Mut/Treat);lm(Y ~ Mut*Treat)
**4B**	None	Linear	Mut(WT,DKO);Treat(Control,Noco)	lm(Y ~ Mut/Treat);lm(Y ~ Mut*Treat)
**4C**	None	Linear	Mut(WT,DKO);Treat(Control,Noco)	lm(Y ~ Mut/Treat);lm(Y ~ Mut*Treat)
**5B**	None	Linear	Mut(WT,DKO,DKO-Serca)	lm(Expr ~ Mut)
**5C**	None	Linear	Mut(WT,DKO,DKO-Serca)	lm(Expr ~ Mut)
**5F**	None	Linear	Mut(WT,DKO,SERCA,BakER);Treat(Control,Noco)	lm(Y ~ Mut/Treat);lm(Y ~ Mut*Treat)
**5G**	None	Linear	Mut(WT,DKO,SERCA,BakER);Treat(Control,Noco)	lm(Y ~ Mut/Treat);lm(Y ~ Mut*Treat)
**6A**	None	Linear	Mut(WT,DKO,DKO-Serca,DKO-BAKret);Treat(Co,Noco48h+4d)	lm(Y ~ Mut/Treat);lm(Y ~ Mut*Treat)
**6B**	None	Linear	Mut(WT,DKO,DKO-Serca,DKO-BAKret);Treat(Co,Noco48h+4d)	lm(Y ~ Mut/Treat);lm(Y ~ Mut*Treat)
**6C**	None	Linear	Mut(WT,DKO,DKO-Serca,DKO-BAKret);Treat(Co,Noco48h+4d)	lm(Y ~ Mut/Treat);lm(Y ~ Mut*Treat)
**6D**	None	Linear	Mut(WT,DKO,DKO-Serca,DKO-BAKret);Treat(Co,Noco48h+4d)	lm(Y ~ Mut/Treat);lm(Y ~ Mut*Treat)
**7B**	None	Linear	Mut(WT,DKO,SERCA,BakER);Chrom(4n,2n)	lm(Y ~ Mut/Chrom);lm(Y ~ Mut*Chrom)
**7D & 7E**	None	Mixed linear	NbSortedCells(10.000cells,50.000cells,100.000cells) or NbSortedCells(1.000cells 2.000cells 5.000cells);Mut(WT,DKO,DKO-Serca,DKO-BAKret);MutReplicate(WTD1,WTD2,WTD3,WTD4,DKOD1,DKOD2,DKOD3,DKOD4,DKOD5,DKO-SercaD1,DKO-SercaD2,DKO-SercaD3,DKO-SercaD4,DKO-SercaD5,DKO-BAKretD1,DKO-BAKretD2,DKO-BAKretD3,DKO-BAKretD4,DKO-BAKretD5,DKO-SercaD6)	lme(Y ~ NbSortedCells/Mut,random = ~ 1|MutReplicate)
**S1C**	None	Linear	Mut(WT,DKO);Treat(Co,MTX1uM,CDDP10uM,OXA50uM,Noco100nM,CytD1.2uM,PCT300nM,TXT100nM,VB4uM)	lm(Y ~ Mut/Treat);lm(Y ~ Mut*Treat)
**S3B**	log10	Linear	Mut(MEF_WT,MEF_DKO);Chrom(2N,4N);Treat(Co,Noc_48h,Noco_48h+4d)	lm(Y ~ (Treat*Chrom)/Mut);lm(Y ~ (Mut*Treat)/Chrom);lm(Y ~ Chrom*(Mut*Treat)
**S3C**	log10	Linear	Mut(MEF_WT,MEF_DKO);Chrom(2N,4N);Treat(Co,Noc_48h,Noco_48h+4d)	lm(Y ~ (Treat*Chrom)/Mut);lm(Y ~ (Mut*Treat)/Chrom);lm(Y ~ Chrom*(Mut*Treat)
**S3D**	log10	Linear	Mut(MEF_WT,MEF_DKO);Chrom(2N,4N);Treat(Co,Noc_48h,Noco_48h+4d)	lm(Y ~ (Treat*Chrom)/Mut);lm(Y ~ (Mut*Treat)/Chrom);lm(Y ~ Chrom*(Mut*Treat)
**S4B**	log10	Linear	Mut(WT,DKO);Treat(Co,Noc48h,Noco48h+4);Chrom(2N,4N)	lm(Y ~ (Mut*Treat)/Chrom,data = DataFigS4ALog);lm(Y ~ Chrom*(Mut*Treat),data = DataFigS4ALog)
**S4C**	log10	Linear	Mut(WT,DKO);Treat(Co,Noc48h,Noco48h+4);Chrom(2N,4N)	lm(Y ~ (Mut*Treat)/Chrom,data = DataFigS4ALog);lm(Y ~ Chrom*(Mut*Treat),data = DataFigS4ALog)
**S4D**	log10	Linear	Mut(WT,DKO);Treat(Co,Noc48h,Noco48h+4);Chrom(2N,4N)	lm(Y ~ (Mut*Treat)/Chrom,data = DataFigS4ALog);lm(Y ~ Chrom*(Mut*Treat),data = DataFigS4ALog)
**S5A**	log10	Linear	Mut(WT,DKO);Treat(Co,48h,48h+4d)	lm(Y ~ Mut/Treat);lm(Y ~ Mut*Treat)
**S5B**	None	Linear	Mut(WT,DKO);Treat(Co,Noco 48h,Noco 48h+4d)	lm(LC3 ~ Mut/Treat);lm(LC3 ~ Mut*Treat);lm(p62 ~ Mut/Treat);lm(p62 ~ Mut*Treat)
