# Supplementary material for: Paradoxical implication of BAX/BAK in the persistence of tetraploid cells
Source: Cell Death Dis. 2021 Nov 1;12(11):1039. doi: 10.1038/s41419-021-04321-3 (PMC8560871; doi:10.1038/s41419-021-04321-3)
Supplement: Supplementary file 9 — Author Contribution Form [file 41419_2021_4321_MOESM9_ESM.pdf]

DECLARATION OF CONTRIBUTIONS TO ARTICLE

ADMC

Manuscript Number: CDDIS-21-2245-R

Journal Name: *Cell Death & Disease* (the 'Journal')

Proposed Title of the Contribution: Paradoxical implication of BAX/BAK in the persistence of tetraploid cells (the 'Contribution')

Author(s): Jiayin Deng, Lucía G. Gutiérrez, Gautier Stoll, Isabelle Martins, Lucía Núñez, Omar Motiño, José Manuel Bravo-San Pedro, Juliette Humeau, Chloé Bordenave, Juncheng Pan, Hélène Fohrer-Ting, Sylvie Souquere, Gerard Pierron, Claudio Hetz, Carlos Villalobos, Guido Kroemer, Laura Senovilla. (the 'Authors')

For all *CDDis* articles, each person named as an author in the published version must be able to show he or she has contributed substantially to the article.

Authorship credit should be based on 1) substantial contributions to conception and design, acquisition of data, or analysis and interpretation of data; 2) drafting the article or revising it critically for important intellectual content; and 3) final approval of the version to be published. Authors should meet conditions 1, 2 and 3.

Any person who cannot be shown to have made a substantial contribution to the article cannot be listed as an author in the final version. The name of any person who is deemed to have made a minor contribution can, however, appear in the Acknowledgments section of the article.

Please complete the table below to indicate the contributions of all named authors to the manuscript.

Author Full Name:	Specification of Contribution to the Manuscript:
Jiayin Deng	Acquisition, analysis and interpretation of data. Revising the article. Final approval of the version to be published.
Lucía G. Gutiérrez	Acquisition, analysis and interpretation of data. Revising the article. Final approval of the version to be published.
Gautier Stoll	Design and interpretation of bioinformatics and statistical analyses. Drafting and revising the article. Final approval of the version to be published.
Omar Motiño	Design, acquisition, analysis and interpretation of data. Conducting the experiments that have been used to answer the Rebuttal Letter. Revising the article. Final approval of the version to be published.
Isabelle Martins	Design, acquisition, analysis and interpretation of data related to flow cytometry. Revising the article. Final approval of the version to be published.
Lucía Nuñez	Design, acquisition, analysis and interpretation of data related to calcium imaging. Revising the article. Final approval of the version to be published.
José Manuel Bravo-San Pedro	Generation of HCT116 DKO cells. Design, acquisition, analysis and interpretation of data. Revising the article. Final approval of the version to be published.
Juliette Humeau	Generation of HCT116 DKO cells. Acquisition, analysis and interpretation of data. Revising the article. Final approval of the version to be published.
Chloé Bordenave	Optimization of experiments and acquisition of data. Revising the article. Final approval of the version to be published.
Juncheng Pan	Acquisition of data. Revising the article. Final approval of the version to be published.
Hélène Fohrer-Ting	Design, acquisition and analysis of data related to clonogenic capacity experiments. Revising the article. Final approval of the version to be published.
Sylvie Souquère	Acquisition, analysis and interpretation of data related to electron microscopy. Revising the article. Final approval of the version to be published.
Gerard Pierron	Analysis and interpretation of data related to electron microscopy. Revising the article. Final approval of the version to be published.

Please complete the table below to indicate the contributions of all named authors to the figures.

Figure 1:

A-J: Jiayin Deng, Juncheng Pan, Gautier Stoll

Figure 2:

A-C: José Manuel Bravo-San Pedro, Juliette Humeau, Chloé Bordenave, Hélène Fohrer-Ting, Gautier Stoll
D: Jiayin Deng, Hélène Fohrer-Ting, Gautier Stoll
E: José Manuel Bravo-San Pedro, Juliette Humeau, Hélène Fohrer-Ting, Gautier Stoll
F: Lucia G. Gutiérrez, Laura Senovilla, Hélène Fohrer-Ting, Gautier Stoll

Figure 3:

A-G: Lucía G. Gutiérrez, Laura Senovilla, Gautier Stoll
H-K: Lucía G. Gutiérrez, Omar Motiño, Gautier Stoll

Figure 4:

A-C: Lucía G. Gutiérrez, Lucía Nuñez, Gautier Stoll

Figure 5:

A-B: Laura Senovilla, Isabelle Martins,Gautier Stoll
C: Jiayin Deng, Gautier Stoll
D: Lucía G. Gutiérrez, Gautier Stoll
E-G: Lucía G. Gutiérrez, Lucía Nuñez, Gautier Stoll

Figure 6:

A-B: Lucía G. Gutiérrez, Omar Motiño, Gautier Stoll
C-D: Omar Motiño, Gautier Stoll

Signed for and on behalf of the Author(s):	Print Name:	Date:
[signature]	CDDIS-21-2245R	September 28th, 2021
